# SEMtree: tree-based structure learning methods with structural equation models

**DOI:** 10.1093/bioinformatics/btad377

**Published:** 2023-06-09

**Authors:** Mario Grassi, Barbara Tarantino

**Affiliations:** Department of Brain and Behavioral Sciences, University of Pavia, Pavia 27100, Italy; Department of Brain and Behavioral Sciences, University of Pavia, Pavia 27100, Italy

## Abstract

**Motivation:**

With the exponential growth of expression and protein–protein interaction (PPI) data, the identification of functional modules in PPI networks that show striking changes in molecular activity or phenotypic signatures becomes of particular interest to reveal process-specific information that is correlated with cellular or disease states. This requires both the identification of network nodes with reliability scores and the availability of an efficient technique to locate the network regions with the highest scores. In the literature, a number of heuristic methods have been suggested. We propose SEMtree(), a set of tree-based structure discovery algorithms, combining graph and statistically interpretable parameters together with a user-friendly R package based on structural equation models framework.

**Results:**

Condition-specific changes from differential expression and gene–gene co-expression are recovered with statistical testing of node, directed edge, and directed path difference between groups. In the end, from a list of seed (i.e. disease) genes or gene *P*-values, the perturbed modules with undirected edges are generated with five state-of-the-art active subnetwork detection methods. The latter are supplied to causal additive trees based on Chu–Liu–Edmonds’ algorithm (Chow and Liu, Approximating discrete probability distributions with dependence trees. IEEE Trans Inform Theory 1968;14:462–7) in SEMtree() to be converted in directed trees. This conversion allows to compare the methods in terms of directed active subnetworks. We applied SEMtree() to both Coronavirus disease (COVID-19) RNA-seq dataset (GEO accession: GSE172114) and simulated datasets with various differential expression patterns. Compared to existing methods, SEMtree() is able to capture biologically relevant subnetworks with simple visualization of directed paths, good perturbation extraction, and classifier performance.

**Availability and implementation:**

SEMtree() function is implemented in the R package SEMgraph, easily available at https://CRAN.R-project.org/package=SEMgraph.

## 1 Background

The biological function on the molecular level emerges from the complex interaction of biological entities of a cell. Specifically, different types of Omics-data can interact in many various ways with each other in dependence on the tissue type and the environmental condition of an organism. The interactions among biological molecules can be broadly categorized into three types of networks: metabolic networks, transcriptional regulatory networks, and protein interaction networks (Vidal *et al.* 2011). These networks need to be inferred from the experimental observations generated by different high-throughput platforms, including next-generation sequencing, proteomics, and microarrays.

The goal is to identify active modules, i.e. subnetworks enriched in interactions and in nodes of interest (showing condition-specific changes). Then, these active modules facilitate the investigation of the perturbed cellular responses, as functional modules are the building blocks of the cellular processes and pathways (Mitra *et al.* 2013). To identify these subnetworks, numerous methods have been suggested. These methods can typically be divided into two categories: responsive subnetwork identification and subnetwork extraction started by seed genes (or nodes).

For the first category, a number of algorithms and tools are created by combining genome-wide measurements of signals with pre-established networks (Ideker *et al.* 2002, Beisser *et al.* 2010, Ma *et al.* 2011). These techniques often include a score function quantifying the alternation of a given subnetwork between different conditions as well as a search strategy that aims to identify the subnetworks in the reference network that have the highest scores. Different scoring functions have imposed scores on network nodes or edges or both. Besides, high-scoring nodes were prioritized as “disease genes” useful for generating new hypothesis (Gu *et al.* 2010, Zheng and Zhao 2012).

In the second category, algorithms typically start with a set of genes as seeds to expand and extract a subnetwork from the reference network. The resultant subnetworks, which reflect the paths in which the seeds are involved, suggest the functional relationships of the seed genes and further predict additional genes that may play important roles in functional cooperation (Kleinberg and Tardos 2006).

This class of methods has two main components: a scoring function quantifying the alternation of a given subnetwork between different conditions, and a search algorithm to extract the highest scoring subnetworks. Different scoring functions have imposed scores on network nodes or edges or both. Besides, high-scoring nodes were prioritized as “seed genes” for searching (Gu *et al.* 2010, Zheng and Zhao 2012). Due to the non-deterministic polynomial-time hard nature of the problem of finding the maximal-scoring connected subgraph, it can only be approached by heuristic or approximate methods. Most approaches rely on greedy searches, simulated annealing, and genetic algorithms [see Mitra *et al.* (2013) and Nguyen *et al.* (2019) for general surveys of the active module identification methods]. Because of the diversity of scoring functions and searching algorithms, it is impossible to obtain identical or similar subnetworks given the same input expression profiles and protein–protein interaction (PPI) network.

The main contribution of this article is the development of a self-contained tree-based structure learning algorithm developed into the framework of structural equation models (SEM), called SEMtree() and included in the R package SEMgraph (Grassi *et al.* 2022). To investigate the utility of our approach, we performed two sets of experiments on both observed and simulated expression data using Human Protein Reference Database interaction network, including 5007 proteins and 42 704 interactions from KEGG database (Kanehisa and Goto 2000). We tested the ability of our framework to evaluate plausible regulatory subnetworks of five popular subnetwork detection methods, i.e. BioNet (Beisser *et al.* 2010), COSINE (Ma *et al.* 2011), pathfindeR (Ulgen *et al.* 2019), WalktrapGM (Petrochilos *et al.* 2013), and our fast Steiner tree (ST) function to provide a meaningful comparison in terms of performance.

Regarding real data analysis, the highest scoring subnetwork from each method has been recovered as undirected network and supplied to causal additive trees (CAT) (Jakobsen *et al.* 2022) algorithm of SEMtree() to be converted in a directed tree. The latter conversion allows to compare the methods in terms of directed active subnetworks.

The remainder of this article is organized as follows. First, we describe the SEMtree() features both in terms of inference procedure and user interface. Then, we outline the experimental setup constructed to evaluate subnetwork detection methods, including the real data application and simulation design. In the end, we provide the results together with the overall discussion.

## 2 Method and implementation

SEMtree() function includes both graph and data-driven algorithms to recover trees, T=(V,E) with *p* nodes (*V*) and p−1 edges (*E*). A tree is an undirected (or directed) graph without cycles with a *unique* path between any two nodes, where a *path* between two nodes (j,k)∈V can be viewed as a sequence of edges that may have either the same or different direction with respect to neighboring connections. The graph method refers to the ST, a tree from an undirected graph that connects “seed” (e.g. disease) with additional nodes in the “most compact” way possible based on a very fast solution provided by the Kou’s algorithm (Kou *et al.* 1981). The data-driven methods propose fast and scalable procedures based on the Chu–Liu–Edmonds’ (CLE) algorithm (Chow and Liu 1968) to recover a tree from a full graph. The first method, called CAT (Jakobsen *et al.* 2022), uses pairwise mutual weights as input for the CLE algorithm to recover a directed tree (*arborescence*). The second one (Lou *et al.* 2021) applies the CLE algorithm for skeleton recovery and extends the skeleton to a *polytree* represented by a completed partially directed acyclic graph (CPDAG). Finally, applying the Prim’s algorithm (Prim 1957), the minimum spanning tree (MST) of a connected undirected graph (or a data-driven undirected full graph) can be identified. Here, we review the novel CAT method used for the conversion of undirected graphs in directed ones.

### 2.1 Causal tree recovery

A fundamental problem is learning the causal structure of a random vector $Y=(Y_{1},Y_{2},\ldots ,Y_{p})$ without the graph knowledge. Generally, a directed acyclic graph (DAG), G=(V,E) is used to understand whether $Y_{k}$ causes $Y_{j}$ (or vice versa), where *V* is the set of nodes (i.e. variables) and *E* is the set of edges (i.e. connections), and loops are not allowed. Causality is evaluated over *directed paths* between two nodes having causal relevance, i.e. a sequence of edges with the same direction, where node $Y_{k}$ is an *ancestor* of $Y_{j}$, and $Y_{j}$ is a *descendant* of $Y_{k}$. If $Y_{k}$ and $Y_{j}$ have a direct link ($Y_{k}\to Y_{j}$), $Y_{k}$ is the *parent* of the *child* $Y_{j}$. A DAG can also be represented as an SEM, with no confounding unobserved variables, as follows: where $Y_{j}$ and $U_{j}$ are an observed variable and an unobserved error term, respectively; pa(j) is the parent set of $Y_{j}$ and $\beta_{jk}$ is the regression coefficient, i.e. the weight of the direct link ($Y_{k}\to Y_{j}$). DAG models assume independent errors (no confounding), $\mathrm{cov}(U_{j};U_{K})=0$, and unequal error variances, $\sigma_{j}=\mathrm{var}(U_{j})$ with a Gaussian (normal) distribution, $U_{j}∼N(0,\sigma_{j})$ for all j∈V.


(1) $$Y_{j}=\sum_{k\;\in \;\text{pa}(j)}\beta_{jk}Y_{k}+U_{j},\text{for all }j\in V$$

For high dimensional data, recently Jakobsen *et al.* (2022) suggest models of reduced complexity (i.e. directed trees) as causal graphs. Their approach is known as CAT. A directed tree is a connected DAG in which all nodes have a unique parent, except the *root node* (*r*) with none parent. The node *r* is the unique node with a directed path to any other nodes in the tree. In graph theory, a directed tree is also called an *arborescence*, a *directed rooted tree*, and a *rooted out-tree*, and is a sub-class of *polytree* that allows multiple root nodes, and nodes with multiple parents. CAT is also a SEM defined with bivariate non-linear structural equations: where $f_{j}(.)$ is a non-linear function of any form between the child $Y_{j}$ and the unique parent $Y_{k}=Y_{pa(j)}$, i.e. ($Y_{k}\to Y_{j}$), and $f_{j}(.)=Y_{k}^{3}$, or $f_{j}(.)=\text{sin}(Y_{k})$, or $f_{j}(.)=Y_{k}+Y_{k}^{2}+Y_{k}^{3}$, etc. While, the additive $U_{j}$ term is assumed with a Gaussian distribution as in linear SEM.


(2) $$Y_{j}=f_{j}(Y_{pa(j)})+U_{j},\text{for all }j\in V$$

Generally, the causal structure is not identifiable from the observational data. Common “data-driven” structure learning methods (Heinze-Deml *et al.* 2018) use different assumptions to ensure identifiability of the causal DAG or a list of all the equivalent DAGs (i.e. a Markov equivalence class) embedded in a CPDAG. The authors (Jakobsen *et al.* 2022) prove that exact identification, and not just an equivalent class, is possible for systems of lesser complexity. CAT procedure consistently recovers the causal directed tree of the non-linear SEM in Equation (2).

**Figure 1.**
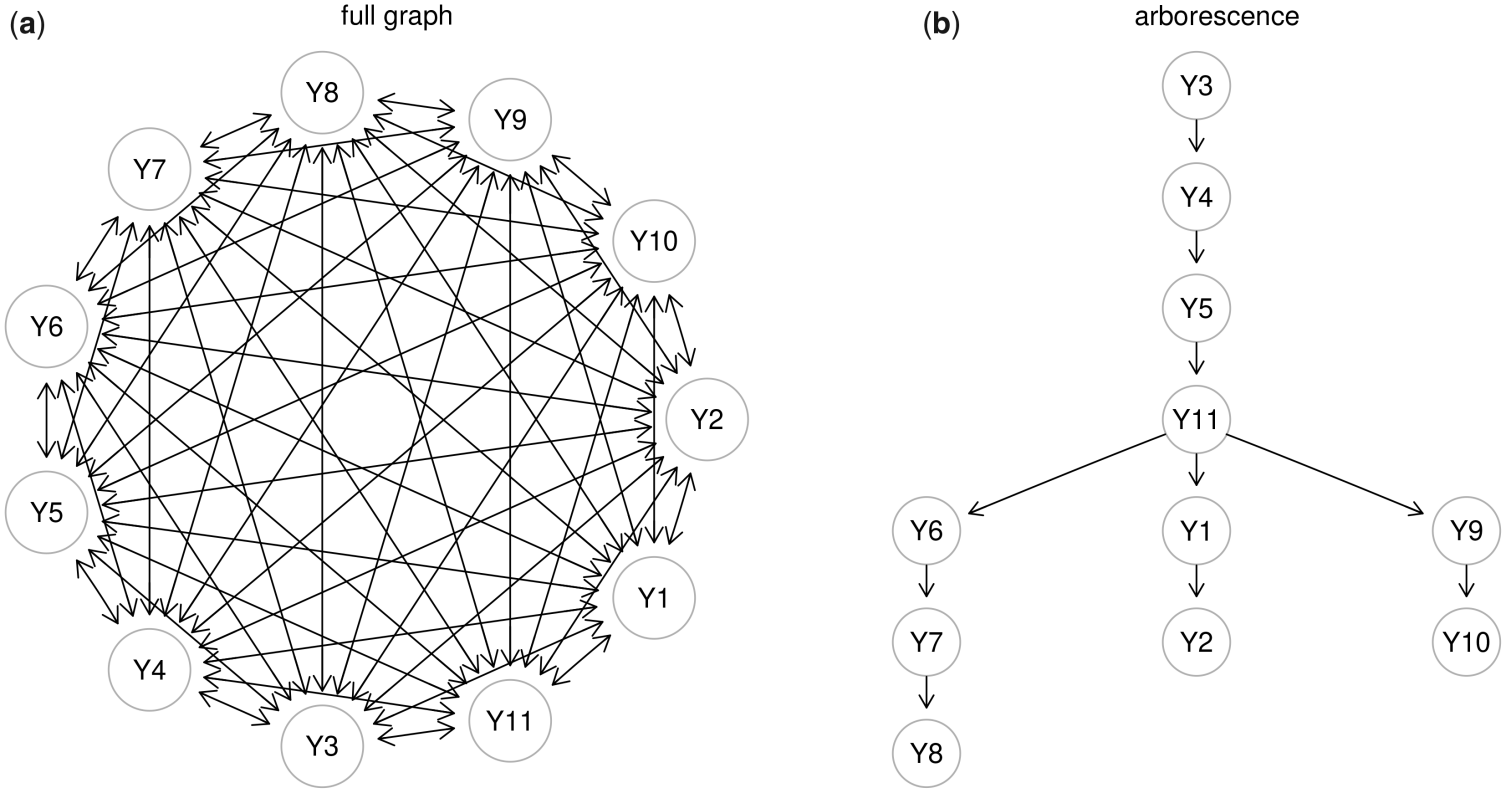
CAT procedure: (a) the fully connected graph with mutual edges and (b) the directed tree (an *arborescence*) minimizing edge weights with CLE’s algorithm, where the edge weights represents the error variance ratio and the lower the value, the better the link prediction.

The causal graph recovery problem (see Fig. 1) is resolved finding a minimum edge weight directed spanning tree of the fully connected graph, G=(V,E) with *p* nodes V=Y and p(p−1) mutual edges $E=(Y_{k}\to Y_{j};Y_{k}\leftarrow Y_{j})$.

CAT uses a score-based method to recover a directed tree, $T=(V,E^{*})$ minimizing a suitable score function, *S* over all mutual edges of the full graph, that is proportional to the Gaussian log-likelihood score function, defined by where $\sigma_{r}$ and $s_{r}$ are the error variance of $U_{j}$ (or $U_{k}$) and the variance of $Y_{j}$ (or $Y_{k}$), respectively. The weight $w_{jk}^{G}$ represents the error variance ratio and the lower the value, the better the link prediction. It is simple to implement, computationally efficient, and only requires two steps. The mutual edge weights of the directed full graph are estimated using the residual variances of $\left(Y_{j}-f_{j}(Y_{k})\right)$ and $\left(Y_{k}-f_{k}(Y_{j})\right)$ from the (bivariate) additive regression methods in the first phase. These weights are then incorporated into the CLE algorithm to recover a directed tree with minimal edge weight in the second phase. To note, the non-linearity is essential to distinguish the links (k→j) and (k←j). In linear regression with standardized variables, the weights are equivalent to the negative mutual information, $-MI=\text{log}[1-\text{abs}(\text{cor}(Y_{j};Y_{k}))]$, a symmetric measure that doesn’t preserve directionality information.


(3) $$S=\min_{T}\sum_{\begin{array}{c} \left(k\leftarrow j\right)\\ \left(k\to j\right) \end{array}}w_{jk}^{G}=\sum \frac{1}{2}\;\text{log}\;\left(\frac{\sigma_{r}}{s_{r}}\right)$$

For the implementation, SEMtree() function performs: (i) additive model fitting with penalized regression splines using the R-function gam from the R-package **mgcv**, in order to obtain estimates of ${\hat{f}}_{j,k}$ (resp. ${\hat{f}}_{k,j}$) and ${\hat{\sigma }}_{j}=\text{var}(Y_{j}-{\hat{f}}_{j,k})$ (resp. ${\hat{\sigma }}_{k}=\text{var}(Y_{k}-{\hat{f}}_{k,j})$) in the weighting phase; (ii) the R-function edmondsOptimumBranching() from the R-package **RBGL** for the CLE algorithm in the recovery phase.

### 2.2 User interface

The example code of the function SEMtree() running CAT is as follows:

SEMtree**(**graph = NULL, data, seed,

**_          _**type = “CAT”, eweight = NULL,

**_          _**verbose = FALSE,*.***)**

The inputs are:

- a *graph* representing the network of interest as *igraph* object or *graph*=NULL, if a full graph is used;
- a gene expression data where rows correspond to subjects, and columns to graph nodes (*data*);
- a vector of user-defined seed nodes (*seed*);
- the Tree-based structure learning method, where four graph and data-driven algorithms are available (*type* = “CAT”, or “CPDAG”, or “ST”, or “MST”);
- the edge weight type for igraph object where by default the edge weights are internally computed using 1-abs(cor), otherwise are determined from the user-defined distances (*eweight*);
- the logical argument *verbose*, if TRUE allows the user to visualize and fitting (through SEMrun() function) the tree.

The output is the recovered tree represented by an *igraph* object. To read more about SEMtree() function, in terms of description and usage, refer to https://rdrr.io/cran/SEMgraph/man/SEMtree.html.

## 3 Experimental design

The workflow of the experimental design is displayed in Supplementary Fig. S1, and we refer to Supplementary Material for additional details.

### 3.1 Benchmark data

Coronavirus disease (COVID-19) RNA-seq expression data from Carapito *et al.* (2022) (GEO accession: GSE172114) have been used as benchmark data with 69 subjects × 14 000 genes. Subjects include patients in the intensive care unit with acute respiratory distress syndrome (“critical group,” *n* = 46) defined as cases, and those in a non-critical care ward under supplemental oxygen (“non-critical group,” *n* = 23) defined as controls. The empirical Bayes technique, as implemented in the **limma** R package (Smyth 2005), was used to fit linear models on the normalized RNA-seq data across the 46 case and 23 control samples. The gene *P*-values were adjusted for multiple testing using the method of Benjamini–Hochberg (BH) (Benjamini and Hochberg 1995). Those *P*-values can be directly used as the input for subnetwork detection, be ranked to select a seed gene set, or be converted into a set of particular weights tailored to the requirement of the model.

Network information has been retrieved from the KEGG interactome object of the SEMgraph package as an igraph network object of 5007 nodes and 44 755 edges corresponding to the union of 225 KEGG pathways extracted using the **ROntoTools** R package (Ansari *et al.* 2017). The latter interactome has been transformed into an *undirected* network to be suitable for fitting the already existing subnetwork detection methods. For efficiency purposes, the network has been filtered according to the genes included in the benchmark data and the largest component has been retained. This procedure results in a reference network of 3033 nodes and 19 735 undirected edges.

### 3.2 Tree (CAT) extraction

The existing subnetwork detection methods (see Table 1 and Supplementary Section S1 for more details) differ for the class of the output in which the recovered active module is represented. Three out of five algorithms, i.e. COSINE, pathfindeR, and WalktrapGM, give as output a list of genes representing the identified subnetworks, not allowing the user to visualize the full graph with the interactions between nodes. On the other side, BioNet and SEMtree() output the subnetwork in an undirected graph format. Therefore, we extract from the obtained gene list of COSINE, pathfindeR, and WalktrapGM the undirected induced subgraphs on the reference undirected KEGG interactome.

**Table 1.** We selected four methods from literature for comprehensive assessment of subnetwork detection if: (i) the method is implemented within a well-maintained R package (or open source R code) and (ii) it represents diversity of methodology.^a^

Method (ref)	Algorithm	Input network	Input data	Node scoring	Edge scoring
BioNet (Beisser *et al.* 2010)	Integer-linear programming	HPRD	*P*-values	*P*-values	
COSINE (Ma *et al.* 2011)	Genetic algorithm	HPRD	Gene expression data	*F*-test	ECF test
pathfindeR (Ulgen *et al.* 2019)	Greedy algorithm	HPRD	*P*-values	*P*-values	
SEMtree (Grassi *et al.* 2022)	Fast ST algorithm (1)	HPRD	Seed	Seed	1-abs(cor)
	Fast ST algorithm (2)	HPRD	Seed	Seed	*r*-to-*z P*-values
WalktrapGM (Petrochilos *et al.* 2013)	Random walk algorithm (1)	HPRD	FC values	FC values	FC values
	Random walk algorithm (2)	HPRD	Gene expression data	*P*-values	*r*-to-*z P*-values

a The table summarizes the selected method, highlighting the key characteristics and the key differences between each method in terms of (i) algorithm used to construct the subnetworks, (ii) input requirements, (iii) node scoring, (iv) edge scoring (if any), and (v) statistical test for assessing the significance of the identified active subnetworks (if any). We selected the ST proposed as default option from SEMtree() function, where edge weights are defined according to 1-abs(cor), and the best performing weights among weightGraph() options, defined by *r*-to-*z P*-values (see Supplementary Section S2 for details on graph weighting procedures and Supplementary Tables S3–S7 for more information about the ST methods’ performance).

Since in Section 3.3 a directed graph structure is required in the benchmark data analysis to evaluate the node perturbation through SEM fitting, the different type of output has been converted to a directed graph (a directed tree) by the following two steps procedure:

1. First, when all the undirected graphs representing the identified active modules have been recovered, their dimensionality has been investigated to have a maximum number of about 200 nodes as the upper bound to retain the interpretability of the recovered modules as suggested by Petrochilos *et al.* (2013), and similar to the size (232) of the KEGG “Coronavirus disease—COVID-19” pathway. Beyond this threshold, to solve this high-dimensionality problem, SEMgraph offers the possibility to merge groups of nodes using hierarchical clustering with prototypes from the **protoclust** R package (Minmax linkage) (Bien and Tibshirani 2011) with mergeNodes() function. We therefore have a single representative data point (the prototype) for the resulting cluster for each merging of the agglomerative procedure. The mergeNodes() function cuts the dendrogram at height $h=1-\text{abs}(\rho_{0}$), where $\rho_{0}$ is the Pearson’s correlation coefficient, cor($Y_{j};Y_{k}$). This procedure results in a merged node (and a reduced graph) in which every node in the cluster has correlation of at least $\rho_{0}$ with the prototype node. We tuned the height *h* to control the size of subnetworks to be approximately 200 genes.
2. Second, after merging nodes, an arborescence layout with CAT algorithm has been recovered from each method to (i) be more comparable from a structural viewpoint with a more interpretable yet visible subnetwork, (ii) to identify gene signature, i.e. significant root node, driver-gene and hub or module structure, and (iii) to reduce considerably the CPU time computation of SEM fitting.

We refer the reader to Supplementary Figs S4–S12 for the visualization of the recovered CAT subnetwork of each method.

### 3.3 Evaluation metrics

In the benchmark data analysis, the performance of the state-of-the-art approaches has been evaluated in terms of (i) system perturbation, (ii) disease classifier performance, and (iii) COVID-19 gene set/GO enrichment. We also add to the seven extracted CAT modules two reference trees (after CAT conversion): (8) the KEGG “Coronavirus disease—COVID-19” pathway, and (9) the data-driven directed tree extracted from the top 200 DEGs ranking by a Random Forest variable importance procedure with the randomForest() function of **randomForest** R package (Breiman 2001).

1. Evaluation of system perturbation of extracted CAT subnetworks has been evaluated via SEMace() and SEMgsa() functions of the SEMgraph (Grassi *et al.* 2022, Grassi and Tarantino 2022). For method comparison, we report (i) the number of significant source–sink paths (*P* <0.05 after BH correction) over the total estimated paths; (ii) the Bonferroni combination of ACEs’ *P*-values ($P=K*\text{min}(p_{1},p_{2},\ldots ,p_{K})$), where *K* is the total estimated paths, the lower the value, the better the score; (iii) the number of DEGs, i.e. differential expression genes with *P*-values <0.05 after BH correction, and (iv) the node activation and node inhibition *P*-values (*P*+ and *P*−, respectively) through a Bonferroni statistics (P=2*min(P+;P−)).
2. Disease classifier performance was carried out by a penalized Fisher’s discriminant analysis (pFDA) with the PenalizedLDA() function of **PenalizedLDA** R package (Witten and Tibshirani 2011) to identify genes in the extracted subnetworks able to discriminate between groups. We highlight (i) sensitivity; (ii) specificity, and (iii) accuracy of the FDA classifier.
3. We perform an assessment of enrichment performance, both on the benchmark and simulated data, looking at precision, recall, and F1-score. To this goal, the genes (or the GO terms) are separated into two groups: foreground genes (FG) (or foreground GO terms, FGO) and background genes (BG) (or background GO terms). The FG (FGO) are the reference 92 COVID-19 genes (1099 GO terms), while, for the simulated data, FG genes are artificially differentially expressed. Then, (i) precision, (ii) recall, and (iii) F1-score have been computed (taking the average over 100 simulation runs for the simulated data).

We refer the reader to Supplementary Fig. S1 for the visualization of the active-subnetwork search approach and to Supplementary Section S3 for more details about the evaluation metrics.

### 3.4 Data simulations

Following the experimental setup of Ma *et al.* (2011), we simulated five datasets, including one “white” dataset (i.e. control) and four datasets to be compared to the control one (i.e. cases) from multivariate normal distributions. Different mean parameters (μ) and covariance matrices (with different ρ correlation coefficient) were set for each dataset, fixing the variances to 1. Each dataset consists of 500 genes and 20 samples and the condition-specific subnetwork for case datasets 1, 2, 3 consisted of 50 genes, while for the case dataset 4 consisted of 40 genes. More details are given in Supplementary Section S3.

Given the PPI network recovered from KEGG database and the ground truth subnetwork, four gene expression data (against one control dataset) were simulated with 100 randomizations. Then we performed differential expression analysis across the 20 case and 20 control samples and we assigned to each gene an adjusted *P*-value representing its significance of differential expression. Gene expression data, DEGs or *P*-values were supplied according to the subnetwork detection method of interest. We ran 6 selected subnetwork methods 100 times for 4 case datasets. Finally, we obtained 2400 (100 randomizations × 4 case datasets × 6 methods) subnetworks. Note that, for each simulation run, the evaluation metrics (average Recall, Precision, and F1-score over 100 runs) have been computed only if an active module with more than one node has been identified.

## 4 Results

### 4.1 Benchmark results

We aim to apply SEMtree() on COVID-19 real data to compare its performance with existing methods and to reveal significant biological processes. The goal is to retrieve a single condition-specific subnetwork composed of genes with a good system perturbation, while reporting optimal ability to discriminate between groups. In addition, the ability of each method to identify COVID-19-related genes (gene enrichment) and GO terms related to those genes (GO enrichment) has been tested.

Table 2 shows that the highest percentage of source–sink path perturbation and the lowest combination of path *P*-values (*ACEs(%)* and *PVAL(E)*, respectively) is reported by ST, in line with RF_C19 and immediately followed by STr2z. pathfindeR reports the most perturbed network, with 112 DEGs (*No.DEGS*) and the lowest combination of node *P*-values *(PVAL(V)*), followed by BioNet, ST, and STr2z. The combination of all these metrics allows to consistently identify ST as the most perturbed subnetworks among the considered ones in terms of both path and node perturbation.

**Table 2.** Evaluation metrics (graph filtering and system perturbation) from the benchmark data analysis.^a^

				System perturbation			
Method	Graph	*h*	Tree	ACEs (%)	PVAL(E)	No. DEGs	PVAL(V)
BioNet	(263; 569)	0.1	(193; 192)	19	2.70e-04	112	2.15e-08
COSINE	(241; 171)	0.2	(206; 205)	2	3.44e-02	57	8.71e-09
pathfindeR	(264; 700)	0.1	(205; 204)	0	2.86e-01	112	2.78e-11
ST	(396; 395)	0.15	(192; 191)	63	5.41e-06	103	4.91e-10
STr2z	(459; 458)	0.2	(204; 203)	22	1.55e-05	94	3.17e-13
WGM_RWR	(166; 600)	0	(166; 165)	0	4.17e-01	66	3.75e-10
WGM_FC	(155; 560)	0	(155; 154)	4	9.64e-02	49	4.77e-08
KEGG_C19	(183; 113)	0	(183; 182)	0	3.17e-01	48	1.64e-10
RF_C19	(200; 199)	0	(200; 199)	43	6.58e-03	141	2.09e-12

a The original graph size (*graph*), the optimal height (*h*) to cut the minimax clustering, and the direct tree (arborescence) structure (*tree*) have been firstly displayed. Then, the path perturbation of each method can be evaluated looking at the percentage of significant paths in the network together with the combination of their *P*-values (ACEs (%) and PVAL(E)_**,**_ respectively). Node perturbation can be measured with the number of DEGs (*No*.*DEGS*) in the network and the combination of node activation and inhibition *P*-values (PVAL(V)).

In addition, Table 3 shows that most of the methods report high accuracy values (above 90%) in classifying patients as case or non-case, with the exception of COSINE and WGM_FC that report accuracy below 90% but still around 80%. However, according to the higher number of zero features (*no.zero*), the most parsimonious predictors (genes) are in STr2z, WGM_RW, WGM_FC, and ST. BioNet reports high classification metrics but almost all the features have non-zero discriminant vector. To note, the reference modules have the poorer (KEGG_C19) and the greater (RF_C19) classification performance.

**Table 3.** Evaluation metrics (disease classifier performance and gene/GO enrichment) from the benchmark data analysis.^a^

	Disease classifier performance					Gene/GO enrichment					
Method	No. genes	No. zeros	Se	Sp	Acc	GenePre	GeneRec	GeneF1	GOPre	GORec	GOF1
BioNet	193	2	0.96	0.87	0.93	0.07	0.14	0.09	0.51	0.4	0.45
COSINE	206	20	0.89	0.87	0.88	0.04	0.09	0.05	0.47	0.17	0.25
pathfindeR	205	42	0.96	0.87	0.93	0.07	0.16	0.1	0.41	0.64	0.50
ST	192	46	0.96	0.87	0.93	0.08	0.16	0.11	0.41	0.49	0.44
STr2z	204	87	0.96	0.87	0.93	0.09	0.2	0.12	0.35	0.59	0.44
WGM_RW	166	59	0.93	0.83	0.90	0.07	0.13	0.09	0.50	0.39	0.44
WGM_FC	155	47	0.91	0.83	0.88	0.03	0.05	0.04	0.53	0.36	0.43
KEGG_C19	183	80	0.80	0.78	0.80	0.17	0.34	0.23	0.62	0.46	0.53
RF_C19	200	0	0.96	0.91	0.94	0.03	0.05	0.03	0	0	NA

a The ability of each method to discriminate between groups has been tested via pFDA and it has been evaluated in terms of number of zero features (*no*.*zeros*, with zero penalized discriminant vector) in relation to the number of recovered genes (*no*.*genes*) and the classical classification metrics (Sensitivity *Se*, Specificity *Sp*, Accuracy *Acc*). In addition, gene and GO precision, recall and F1-score are also reported (*GenePre*, *GeneRec*, *GeneF*1, *GOPre*, *GORec*, *GOF*1).

Gene and GO precision, recall, and F1-score are also shown in Table 3. ST methods show the best performance in identifying COVID-19-related genes, with the highest gene F1-score (0.12 for STr2z and 0.11 for ST) among all the considered methods. The latter methods are able to identify, respectively, 18 and 15 reference genes (see Supplementary Table S1) over the total of 92. ST gene enrichment metrics are in line with KEGG_C19 baseline that reports a gene F1-score equal to 0.23. On the other side, pathfindeR reports the highest GO F1-score equal to 0.50, immediately followed by ST, STr2z, and WGM_RW (0.44). pathfindeR is able to recover 703 reference GO terms over the total of 1099, while STr2z and ST select, respectively, 650 and 535 COVID-19 GO terms. Worst performance, both on gene and GO metrics, is reported by COSINE, with a gene F1-score of 0.05 (with a number of selected COVID-19 genes equal to 8) and a GO F1-score of 0.25 (with a number of selected COVID-19 GO terms equal to 184).

In the end, to better explore the similarity between the seven recovered subnetworks, Jaccard similarity indices (J(A,B)=|A∩B|/|A∪B|, where A∩B| is the intersection of sets *A* and *B* and |A∪B| is the union) have been reported in Supplementary Table S2, excluding ST and WGM_FC for the obvious similarity with STr2z and WGM_RWR. Similarity coefficient around 0.3 is observed by pathfindeR with BioNet, and STr2z, while the other methods seem to have recovered different network structures.

**Figure 2.**
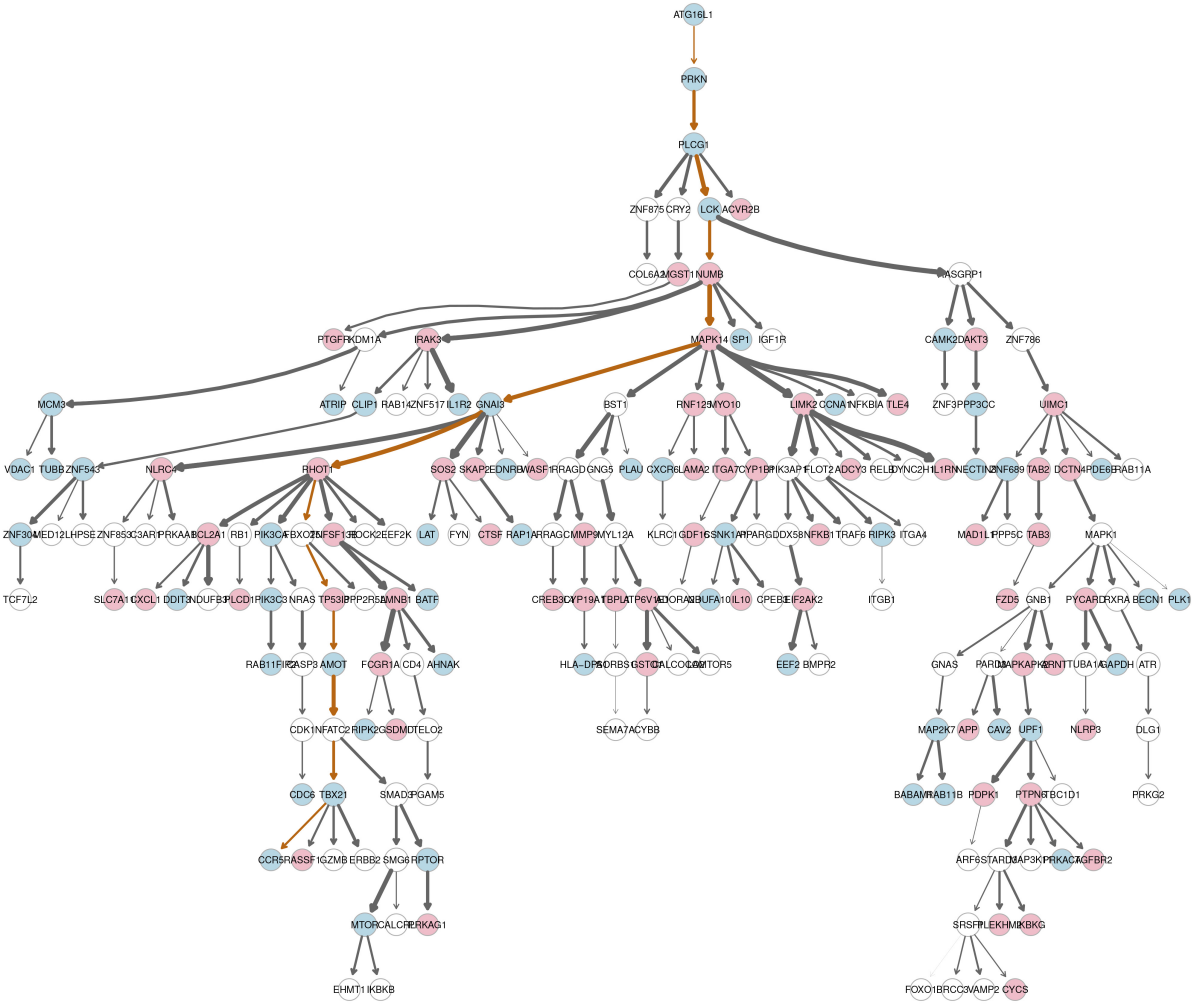
The graph shows 61 differentially activated nodes and 52 differentially inhibited nodes, showing significant variation in the two COVID-19 groups. The remaining 80 (white-shaded) nodes are not differentially regulated. The width of edges shows the strength of correlation coefficient of pairs of interacting nodes. The path between source node **ATG16L1** (Gene ID: 55054) and sink node **CCR5** (Gene ID: 1234) can be highlighted as a significant perturbation route in the disease of interest. The node ATG16L (down-regulated) gene produces a key autophagy protein that interacts with ATG5 and ATG12 to form a complex necessary for the extension of the autophagophore. Through influencing multiple components of the immune response, autophagy plays a crucial antiviral function in a variety of human illnesses (Ahmad *et al.* 2018; Tao *et al.* 2020). However, some viruses, including SARS-CoV-2, have learned how to manipulate the autophagy machinery in order to avoid their destructive destiny. On the other side, CCR5 (down-regulated) is a receptor for proinflammatory chemokines, which are implicated in host responses, particularly to viruses. Findings of Čizmarević *et al.* (2021) imply that the CCR5-32 allele may be protective against SARS-CoV-2 infection and HIV infection alike and represent a predictive biomarker for COVID-19 susceptibility, severity, and death. The activity of three hub structures along the path MAPK14→GNAI3→RHTO1 are altered. According to recent research reports, MAPK14 (up-regulated) stimulates regulation of inflammation that may contribute to exacerbate organ damage linked with complications of COVID-19 (Su *et al.* 2021), GNAI3 (down-regulated) is a gene target predicting COVID-19—hypertension comorbidity pathway crosstalk (Barh *et al.* 2021), and RHTO1 (up-regulated) maps a hub protein sharing interactions with both viral baits and host baits for antiviral drug discovery (Liu *et al.* 2021).

Overall, SEMtree() Kou’s ST algorithm is able to retrieve the subnetwork of interest, with good enrichment metrics, if compared to the other methods. The module retrieved by ST together with its perturbation is reported in Fig. 2. For tree interpretation, the SEMtree() recovered subnetwork can be investigated to identify significant causal paths and hub-genes with high level of graph arborescence, i.e. many edges point away from that specific node. After testing for significant ACEs (*P* <0.05 after Bonferroni correction, see Supplementary Table S8), a significant path consisting of 14 nodes (with only two genes not perturbed) and 13 edges (with high pairwise correlation) between source node **ATG16L1** (Gene ID: 55054) and sink node **CCR5** (Gene ID: 1234) has been found and compared with COVID-19 literature in the legend of Fig. 2. This perturbed route, along with others, between the virus and the host cell interaction could suggest a possible mechanism of viral pathogenesis.

In summary, trees (arborescences) are simple models, but can nevertheless provide useful biological insights and extract unrevealed knowledge-based network structures to experimentally validate new hypothesis for disease (here, COVID-19) research.

### 4.2 Simulation results

To test the seven subnetwork detection methods on the simulated data, each of the four case datasets was compared with the Control Group to identify condition-specific subnetworks. The goal is to retrieve a single condition-specific subnetwork composed of 50 genes, while for the case dataset 4 consisted of 40 genes. Simulation results are shown in Supplementary Fig. S14.

Compared with the other methods, SEMtree() ST and STr2z achieve high precision, around 90%−80% for all the case datasets, just below the precision of BioNet. Since BioNet recovers the smallest subnetwork for all the case datasets (see Supplementary Fig. S13), its precision is the highest one compared to the other methods. SEMtree() recovers the smaller subnetworks immediately after BioNet and, therefore, it shares similar precision metrics with the latter. The highest network dimension is reported by WGM_RWR and WG_FC, resulting in the lower precision scores since the method selected more BG (i.e. false positives). Similar performance is reported by pathfindeR.

Looking at the recall metrics (Supplementary Fig. S14), COSINE reports slightly higher results given that the higher dimensionality of its modules allows to select more genes and obtain a smaller number of false negatives. The recall values of ST and STr2z are in line with BioNet and higher than pathfindeR, WGM_RWR, and WGM_FC.

Then, we calculated the F1-score to determine how good the methods are to retrieve the FG while avoiding picking BG. The F1-score for COSINE is around to 60% for all case datasets, while it is near 30%−40% for ST, STr2z, and BioNet. The latter methods are able to reach the highest F1-scores for case dataset 1 and 3, driven by the high precision values. In detail, STr2z reports F1-score around 60% for case dataset 1 and 3. For more details about simulation metrics, we refer the reader to Supplementary Table S9.

## 5 Discussion

The key challenge in many disciplines is to derive networks from high-dimensional data, and numerous methods have been proposed. Despite being too simple for accurate representations of complex biological processes, trees (undirected and directed) can be used as the starting point to provide a general comprehension of the dependence structure of the network. Directed trees is an obvious choice for causal inference in high-dimensional data. Moreover, we can consider certain attributes of the chosen tree to be substitutes for related attributes of the real, underlying network. Connectivity, path length, and degree are a few attributes that can be employed in this way. All of these factors led us to design SEMtree(), a tree-based structure learning algorithm based on SEM. The ST approach has been chosen to be compared to the other existing methods, representative of the main algorithms dedicated to the identification of active modules: PCST (BioNet), genetic algorithm (COSINE), greedy algorithm (pathfindeR), and random walk (WalktrapGM). We have performed a comprehensive assessment of those subnetwork detection methods using COVID-19 real data and simulation data. The key conclusion in this study can be summarized as follows.

First, based on the real ans sumulation datasets, each of the approaches was asserted to be efficient in their original articles. Our results on benchmark data show high system perturbation for the ST of SEMtree(), while high levels of GO enrichment are reported by pathfindeR. Simulation results report high precision value for BioNet and ST, but a good F1-score around 60% for COSINE. However, worst performance on the benchmark data is reported by COSINE. As none of the methods outperformed other methods overall, users should choose an appropriate method based on the purposes of their studies.

Second, in terms of ease of use, some of the methods do not offer user-friendly interface or visualization functions for the identified subnetworks. Most of the existing subnetwork detection methods output a list of genes representing the module, not allowing the user to visualize the entire network. BioNet outputs the subnetwork in an undirected graph format.

We propose SEMtree() algorithm in order to overcome some limitations of existing literature. The advantages of our algorithm are summarized as follows:

1. SEMtree() function includes four tree-based structure learning methods implemented with graph and data-driven algorithms. Fast Kou’s algorithm has been chosen for comparison with the other existing methods based on the pre-established networks (interactomes), with default edge weighting, but the users can choose one of the methods of weightGraph() function based on their needs (see Supplementary Section S2).
2. SEMtree() utility goes beyond subnetwork detection with the graph extraction functionality. Starting from a seed list, SEMtree() allows the user to recover the structure of the network with data-driven algorithms. In detail, the CAT (arborescence) or the CPDAG (polytree) can be recovered from a user-defined gene list or a list of differentially regulated genes, active modules, or pathways.

In addition, SEMgraph package provides a set of utilities that have been crucial to build up the analysis of the article. These functions allow the user to: cluster the graph (mergeNodes()); apply SEM-based gene set analysis to recover the perturbation metrics (SEMgsa()), evaluate ACEs between source–sink pairs (SEMace()), evaluate SEM fitting given the recovered network and the data of interest (SEMrun()), and visualize the identified module with gplot() function, specifying different type of layouts, and other functions illustrated in Grassi *et al.* (2022). As, to our knowledge, no existing method is able to fully leverage the network and data information as SEMtree(), allowing the user to easily recover the tree-based structure with different algorithms, extract a directed graph from a seed list and visualize the recovered module.

Given the advance in tree development, our direction for future work is also to consider the most recent proposals suggested in finance literature (Ahelegbey *et al.* 2019, Agosto *et al.* 2020, Giudici and Polinesi 2021), and in machine learning (Chatterjee and Vidyasagar 2022, Tramontano *et al.* 2022). Specifically, the random matrix theory (Giudici and Polinesi 2021), and the new xi-coefficient of correlation (Chatterjee and Vidyasagar 2022) could be incorporated in SEMtree() as first-step filtering technique for ST and MST, and as asymmetrical edge scoring in high-dimensional (n<p) regime for CAT, respectively.

## 6 Conclusions

We have shown that SEMtree() is easily accessible to common users and provides robust results under several experimental conditions. It recovers the tree-based structure starting from the interactome and gene expression information while offering good enrichment metrics, perturbation extraction, and classifier performance.

Even though trees are overly simplistic representations of biological systems, we believe that SEMtree() can be a valuable tool for practitioners, not only when undertaking complex subnetwork detection analysis, but also when extracting dependence (causal) structure with a direct tree (arborescence) starting from a list of genes. This simple graph can be useful as a preliminary step for visualizing observational high-dimensional data, highlighting densely connected hub nodes or neighborhoods that might be further investigated.

## Availability of source code and requirements

Project name: SEMtree()(SEMgraph package)

Project home page: https://github.com/fernandoPalluzzi/SEMgraph

Operating system(s): Platform independent

Programming language: R

License: GNU General Public License version 3 or higher (GPL ≥ 3)

Restrictions for non-academic use: None

## Supplementary Material

btad377_Supplementary_Data

## Data Availability

Code to reproduce all results of the analysis, together with the COVID-19 data used in this study can be found in the supplementary files available at: https://github.com/fernandoPalluzzi/SEMgraph/tree/master/SEMtree. All additional information and results about the analysis are listed in Supplementary Material.
