# Supplementary material for: SEMtree: tree-based structure learning methods with structural equation models
Source: Bioinformatics. 2023 Jun 9;39(6):btad377. doi: 10.1093/bioinformatics/btad377 (PMC10287946; doi:10.1093/bioinformatics/btad377)
Supplement: btad377_Supplementary_Data [file btad377_supplementary_data.pdf]

Supplementary material for "SEMtree: tree-based structure learning methods with Structural Equation Models" by Grassi M and Tarantino B

Contents

1 Subnetwork detection methods **1**

2 Graph-weighting methods of the `weightGraph()` function **3**

2.1 SEM (regression) model 3

2.2 Covariance model 4

2.3 Confirmatory Factor Analysis (CFA) model 5

2.4 Fisher's transformation z-to-r correlation method 5

3 Evaluation metrics **6**

List of figures

1 **Overview of the active-subnetwork search approach**. The required input is a gene expression data (n subjects x p genes) or a two-column table representing Gene identifiers and adjusted p-values or log-fold changes associated with differential expression data. The data are then used for active subnetwork search, starting from a KEGG PPI consisting of 3033 nodes and 19735 undirected edges. The active module is then recovered in a network format or a list of genes representing the module. Next, the identified active subnetworks are filtered according to the following steps: 1) an undirected graph is first obtained for each method; 2) group of nodes have been merged according to hierarchical clustering with prototypes; 3) an aborescence layout has been recovered with CAT algorithm. In the end, the evaluation of subnetwork detection methods has been summarized in a table in terms of: 1) system perturbation; 2) disease classifier; 3) gene/GO enrichment. 1

2 **SEM model.** 4

3 **CFA model.** 5

4 **BioNet recovered subnetwork from benchmark data analysis.** Nodes in the recovered subnetworks are coloured in yellow if they represent COVID-19 related genes while the cluster summarised by the prototype is coloured in orange if it contains at least one COVID-19 related gene (in green otherwise). The width of edges shows the strength of correlation coefficient of pairs of interacting nodes. 8

5 **COSINE recovered subnetwork from benchmark data analysis.** Nodes in the recovered subnetworks are coloured in yellow if they represent COVID-19 related genes while the cluster summarised by the prototype is coloured in orange if it contains at least one COVID-19 related gene (in green otherwise). The width of edges shows the strength of correlation coefficient of pairs of interacting nodes. 9

6 **pathfindeR recovered subnetwork from benchmark data analysis.** Nodes in the recovered subnetworks are coloured in yellow if they represent COVID-19 related genes while the cluster summarised by the prototype is coloured in orange if it contains at least one COVID-19 related gene (in green otherwise). The width of edges shows the strength of correlation coefficient of pairs of interacting nodes. 10

7 **ST recovered subnetwork from benchmark data analysis.** Nodes in the recovered subnetworks are coloured in yellow if they represent COVID-19 related genes while the cluster summarised by the prototype is coloured in orange if it contains at least one COVID-19 related gene (in green otherwise). The width of edges shows the strength of correlation coefficient of pairs of interacting nodes. 11

8 **STr2z recovered subnetwork from benchmark data analysis.** Nodes in the recovered subnetworks are coloured in yellow if they represent COVID-19 related genes while the cluster summarised by the prototype is coloured in orange if it contains at least one COVID-19 related gene (in green otherwise). The width of edges shows the strength of correlation coefficient of pairs of interacting nodes. 12

9 **WalktrapGM (with iPINBPA weights) recovered subnetwork from benchmark data analysis.** Nodes in the recovered subnetworks are coloured in yellow if they represent COVID-19 related genes while the cluster summarised by the prototype is coloured in orange if it contains at least one COVID-19 related gene (in green otherwise). The width of edges shows the strength of correlation coefficient of pairs of interacting nodes. 13

10 **WalktrapGM (with FC weights) recovered subnetwork from benchmark data analysis.** Nodes in the recovered subnetworks are coloured in yellow if they represent COVID-19 related genes while the cluster summarised by the prototype is coloured in orange if it contains at least one COVID-19 related gene (in green otherwise). The width of edges shows the strength of correlation coefficient of pairs of interacting nodes. 14

11 **KEGG "Coronavirus disease - COVID-19" pathway" recovered subnetwork from benchmark data analysis.** Nodes in the recovered subnetworks are coloured in yellow if they represent COVID-19 related genes while the cluster summarised by the prototype is coloured in orange if it contains at least one COVID-19 related gene (in green otherwise). The width of edges shows the strength of correlation coefficient of pairs of interacting nodes. 15

12 **Random forest COVID-19 recovered subnetwork from benchmark data analysis.** Nodes in the recovered subnetworks are coloured in yellow if they represent COVID-19 related genes while the cluster summarised by the prototype is coloured in orange if it contains at least one COVID-19 related gene (in green otherwise). The width of edges shows the strength of correlation coefficient of pairs of interacting nodes. 16

13 **Average size of the recovered subnetwork for each method on simulated data.** Nodes in the recovered subnetworks are coloured in yellow if they represent COVID-19 related genes while the cluster summarised by the prototype is coloured in orange if it contains at least one COVID-19 related gene (in green otherwise). The width of edges shows the strength of correlation coefficient of pairs of interacting nodes. 17

14 **Precision, recall and F1-score on simulated data.** The mean over 100 simulation runs of precision, recall and F1- score are displayed for each method and for each case dataset. Results show high precision score for ST and STsem, just below the precision of BioNet. 18

List of tables

1 **Recovered genes/GO and selected COVID-19 related genes/GO for the nine recovered subnetworks from benchmark data analysis.** 19

2 **Jaccard similarity indices between the node sets of the nine recovered subnetworks from benchmark data analysis.** 19

3 **Gene enrichment metrics for all ST methods on benchmark data analysis.** 19

4 **GO enrichment metrics for all ST methods on benchmark data analysis.** 19

5 **Perturbation metrics for all ST methods on benchmark data analysis.** 19

6 **Disease classifier performance metrics for all ST methods on benchmark data analysis.** 20

7 **Jaccard similarity indices between the node sets for all ST methods on benchmark data analysis.** 20

8 **Significant average causal effects (ACEs) between source-sink pairs as obtained from SEMace function while testing for perturbation with SEMpath.** 20

9 **Average simulation results (over 100 runs) for the seven subnetwork detection methods for each case dataset.** 21

1 Subnetwork detection methods

Workflow of the active-subnetwork search approach is display in Figure 1. We selected four methods from literature for comprehensive assessment of subnetwork detection if: (i) the method is implemented within a well-maintained R package (or open source R code) and (ii) it represents diversity of methodology.

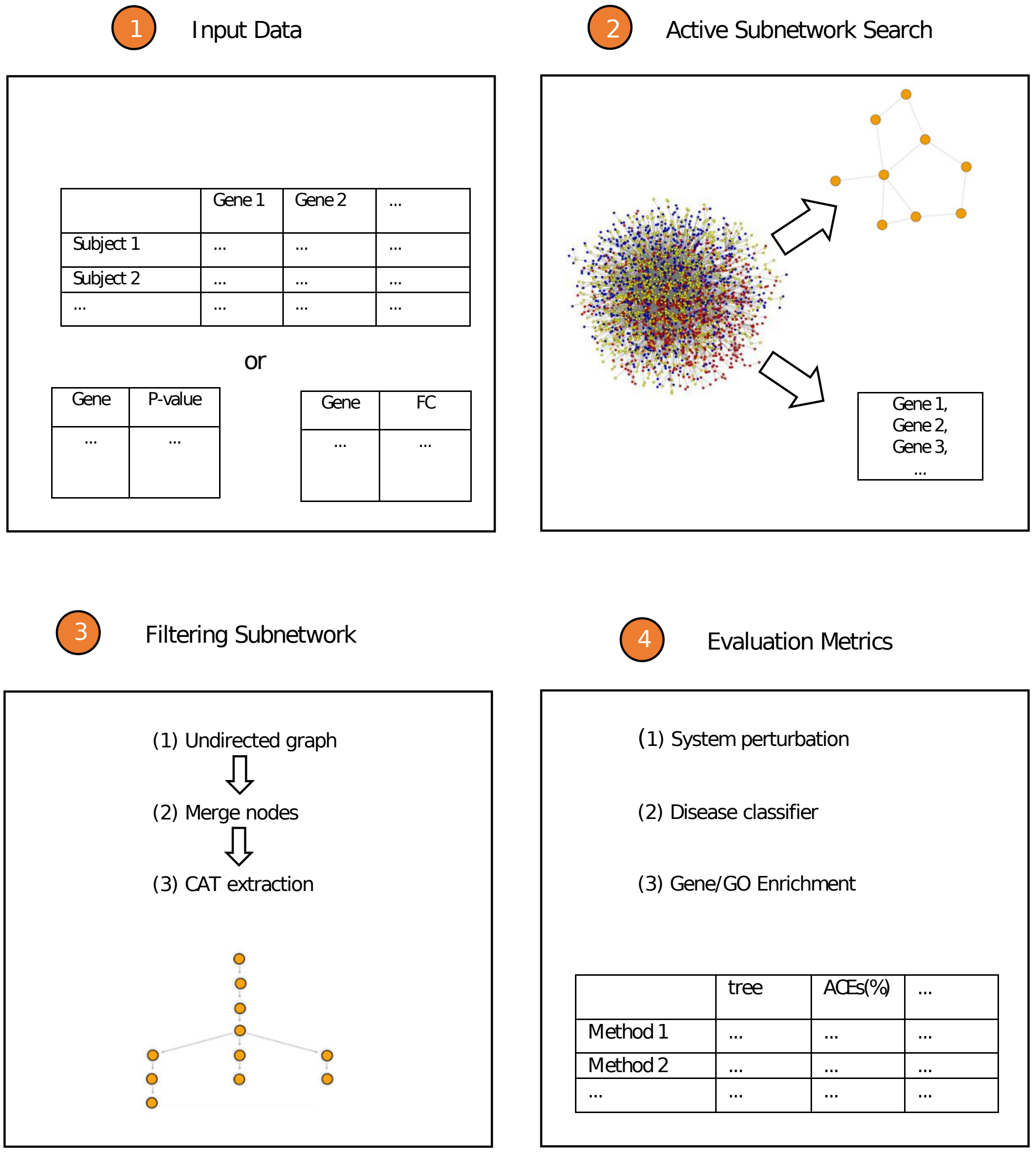

Figure 1: **Overview of the active-subnetwork search approach**. The required input is a gene expression data (n subjects x p genes) or a two-column table representing Gene identifiers and adjusted p-values or log-fold changes associated with differential expression data. The data are then used for active subnetwork search, starting from a KEGG PPI consisting of 3033 nodes and 19735 undirected edges. The active module is then recovered in a network format or a list of genes representing the module. Next, the identified active subnetworks are filtered according to the following steps: 1) an undirected graph is first obtained for each method; 2) group of nodes have been merged according to hierarchical clustering with prototypes; 3) an aborescence layout has been recovered with CAT algorithm. In the end, the evaluation of subnetwork detection methods has been summarized in a table in terms of: 1) system perturbation; 2) disease classifier; 3) gene/GO enrichment.

Table 1 (in the article) summarizes the selected method, highlighting the key characteristics and key differences between each method in terms (i) algorithm used to construct the subnetworks, (ii) input requirements (iii) node scoring, (iv) edge scoring (if any) and (v) statistical test for assessing the significance of the identified active subnetworks (if any).
The methods analyzed here use a wide range of scoring functions to score the nodes and edges. Most of them provide a scoring function for nodes or edges, but only some of them take into account the scores of both nodes and edges. Edge-based scoring networks focus on the strength of the interaction between proteins or genes, whereas node-based scoring networks look at the relevance of one gene or protein in the context of the entire network. Node-based scoring algorithms may yield subnetworks with high scoring nodes but no significant connectivity between nodes. Edge-based scoring network may generate subnetworks with highly related genes but low network relevance. Methods that consider both node and edge scores are more likely to yield a more accurate active module. In principle, scoring functions should be the summary statistics that capture the network perturbation, signal propagation as well as the changes between different phenotypes. Specifically:

- *Steiner tree* (ST) extracts a active subgraph from the input graph such that additional nodes (called the *Steiner* nodes) connecting "seed" nodes (called the *terminal* nodes) minimize the sum of the weight of every edge in the subgraph. ST have been tested with `SEMtree()`, that use the simple but effective heuristic approach based on the fast Kou's algorithm, as first published in 1981 by Kou *et al.* (1981). We used `weightGraph()` from **SEMgraph** package to edge scoring the input graph, specifying different edge weighting methods. For comparison on benchmark data, we select the argument type="ST" proposed as default option from `SEMtree()` function, where edge weights are defined according to 1-abs(cor), and the best performing ST among `weightGraph()` options, resulting in edge weights defined by Fisher's $r - to - z$ method (Fisher, 1915). See sec. 2 of Supplementary Material for details on graph weighting procedures, and Table 4-8 of Supplementary Material for more information about ST selection procedure. The seeds were selected with a False Discovery Rate (FDR) = 5-E06.
- *BioNet* (Dittrich *et al.*, 2008), model the Prize-collecting Steiner tree (PCST) algorithm as a mathematical programming-based optimization that aims to find subgraphs of maximum weight using CPLEX library (a fast heuristic version in R is also implemented in the function `runFastHeinz ()`). BioNet requires multiple nominal (not adjusted) P-values derived from various sources (differential expression analysis, survival analysis, etc) and then models their combination as a Beta distribution, developing an additive scoring, where positive values are statistically significant with a FDR defined by user, and negative otherwise. Here, we use P-values derived from one source and with the threshold for FDR = 5-E06.
- *COSINE* (Ma *et al.*, 2011)), i.e., COndition SpecIfic subNEtwok, on the other hand, uses a Genetic Algorithm (via the function `ga.bin()` in the R package **genalg**) to search for an optimal subnetwork with the highest aggregate score that jointly measures the condition-specific changes of both nodes and edges using F-statistic and Expected Conditional F-statistic (ECF-statistic), respectively. The subnetwork scoring is defined as a weighted average of nodes and edges with the parameter, λ. Thus, this method requires gene expression data as input to evaluate both the differential expression of individual genes and the differential correlation of gene pairs. Here, we use the default parameters of the genetic algorithm, and λ=0.5, i.e., equal relevance for nodes and edge weights.
- *pathfindeR* (Ulgen *et al.*, 2019) identifies active sub-networks in an unweighted reference PPI network by implementations of a greedy algorithm, a simulated annealing algorithm, or a genetic algorithm. Here, we use the greedy algorithm, a problem-solving/optimization procedure that selects locally the best option in each stage with the expectation of reaching the global optimum. The procedure start with a seed node and adds direct neighbors (d=1 by default) in each step to maximize the subnetwork score, ad so on for all seed nodes, removing a subnetwork that overlaps with a higher scoring subnetwork (at 0.5 threshold by default). Here, we use the default parameters, and the non overlapping genes list in the all extracted sub-networks is recovered.
- *WalktrapGM* (Petrochilos *et al.*, 2013) runs a short random-walk-based community detection algorithm to identify disease modules from an edge weighted reference network (via the function `cluster_walktrap()` in the R package **igraph**). According to the node and edge weighting scheme, two different WalktrapGM algorithms have been tested. *WGM_FC* assigns gene fold change (FC) values and estimates the edge weights as a function of differential expression, taking the mean of the absolute FC-values of the two adjacent nodes of the edge (Petrochilos *et al.*, 2013). *WGM_RWR*, a modified R function of *WalktrapGM*, assigns gene p-values to node weights and computes edge weights from Fisher's r-to-z transform for testing pairwise correlation coefficient of interacting nodes. Then, for both algorithms (WGM_FC, and WGM_RWR), module scores are used to rank high-scoring modules, comparing the module cumulative

activity, i.e., the sum of node weights, against a bootstrap distribution of random differential expression values per module size. Here, the first top modules for both algorithms are recovered.

Most of the methods included in this analysis (Steiner Tree, BioNet, pathfindeR, and WalktrapGM) require the user to input a gene list (i.e., a seed list) as the significant gene set or gene P-values to serve as starting points of the algorithm. The only exceptions is COSINE that uses gene expression data and internally computes the F-test and ECF-statistic to capture node and edges changes across multiple conditions. `SEMtree()` allows the user to choose between different types of edge weights for the ST algorithm. To note, pathfindeR and WalktrapGM algorithms require node weights for ranking the sub-networks. Both followed the scoring scheme that was proposed by Ideker et al (Ideker *et al.*, 2002). pathfindeR and WGM_FC use the unweighted sum of node z-score (i.e., the standard normal inverse of a single gene's P-value) adjusted for the size of the sub-network, and calibrated by the mean and standard deviation of a Monte Carlo simulation for each possible sub-network size. WGM_RWR applies the weighted sum of node z-score from iPINBPA (Wang *et al.*, 2015). The weights of the z-scores are obtained with a random walk with restart (RWR) method (Köhler *et al.*, 2008) to prioritize disease-associated genes, and improve sub-network extraction.

2 Graph-weighting methods of the `weightGraph()` function

The input interactome, $G(V,E)$ can be converted into a weighted "perturbed" network, $G(V_W,E_W)$ endowed with nodes and edges weight reflecting their perturbation status. Genes (nodes) can be weighted by bivariate P-values testing the group (1= experimental, 0=control) effect on each gene with t-test o similar tests for two-group differences (via **limma**, **SAM**, etc. in R/Bioconductor packages), or a binary "seed" attribute (1=seed, 0=non-seed) can be associated on each node, if P-value < alpha. As per node weights, gene-gene interactions (edges) can be weighted based on the group difference of pairwise correlation measures.
In **SEMgraph** with the `weightGraph()` function, four trivariate procedures can be performed based on: (i) SEM (regression) model, (ii) Covariance model, (iii) Confirmatory Factor Analysis (CFA) model, and (iv) Fisher's transformation z-to-r correlation method, as follow.

2.1 SEM (regression) model

The SEM (regression) model implies testing the group effects on the source-sink link. A common group effect model of C = {0: control; 1: case} is fitted (see Figure 1) on the source node k, and the sink j:

$$Y_k = \beta_{k0} + \beta_{\mathrm{kC}} C + U_k$$

$$\beta_{j0} + \beta_{jC} C + \beta_{jk} Y_k + \beta_{jkC} Y_j C + U_j$$

or splitting the groups:

$$Y_{k|C=0} = \beta_{k0} + U_j$$

$$Y_{k|C=1} = \beta_{k0} + \beta_{kC} C + U_k$$

$$Y_{j|C=0} = \beta_{j0} + \beta_{jk} Y_k + U_j$$

$$Y_{j|C=1} = (\beta_{j0} + \beta_{jC}) + (\beta_{jk} + \beta_{jkC}) Y_k + U_j$$

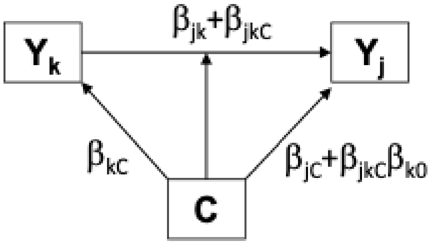

Figure 2: **SEM model.**

the coefficient, β_{kC} represents the intercept (mean) difference between groups in C for source k, while the coefficients, β_{jC} and β_{jkC} represent intercept and slope differences between group $C = 1$, equal to $(\beta_{j0} + \beta_{\mathrm{jC}})$ and $(\beta_{\mathrm{jk}} + \beta_{\mathrm{jkC}})$, and group $C = 0$, equal to β_{j0} and β_{jk}, respectively.

Through causal inference framework, we define two group effect indices (a_1 and a_2), measuring node perturbance (or disease relevance), i.e., the causal total effect (TE) of C on sink j:

$$a_{1j} = (\beta_{jC} + \beta_{jkC} \cdot \beta_{k0}) + \beta_{kC} \cdot (\beta_{jk} + \beta_{jkC})$$

the causal TE, equal to direct effect (DE), of group, C on source k:

$$a_{2k} = \beta_{kC}$$

and a weighted sum defines the new parameter, w combining the TEs of group, C on source and sink nodes:

$$w_{jk} = \mathrm{abs}(a_{ij})/d_j + \mathrm{abs}(a_{2k})/d_k$$

where d_k and d_j are the outgoing degrees (i.e., the number of all direct outgoing connections in the input graph, for each node) of the k-th sources and j-th sinks, respectively. The P-values are computed through the z-test = w/SE(w) of the combined TE of the group effect on the source node j and the sink node k. Of note, the SE(w) is obtained by lavaan syntax specifying w with the ":=" operator.

2.2 Covariance model

The covariance model implies testing the group effects at the same time on the source j, the sink k and their interaction (j, k). Two-group covariance model with an intercept parameter is fitted:

$$Y_j^{(0)} = \alpha_j + U_j^{(0)};\ \ Y_k^{(0)} = \alpha_k + U_k^{(0)};\ \ \mathrm{cov}\left(Y_j^{(0)}; Y_k^{(0)}\right) = \phi_{jk}$$

$$Y_j^{(1)} = \alpha_j + U_j^{(1)};\ \ Y_k^{(1)} = \alpha_k + U_k^{(1)};\ \ \mathrm{cov}\left(Y_j^{(1)}; Y_k^{(1)}\right) = \psi_{jk}$$

A weighted sum defines the new parameter, w combining the group effect on source node (mean difference, $\beta_k - \alpha_k$), sink node (mean difference, $\beta_j - \alpha_j$), and source-sink link (correlation difference, $\phi_{jk} - \psi_{jk}$):

$$w_{jk} = \mathrm{abs}\left(\beta_j - \alpha_j\right)/d_j + \mathrm{abs}\left(\beta_k - \alpha_k\right)/d_j + \mathrm{abs}\left(\phi_{jk} - \psi_{jk}\right)$$

where d_k and d_j are the degree (number of all direct connections in the input interactome, G) of the source k and sink j nodes. P-values are yielded by the t-test=w/SE(w) on the combined difference of the group over the source node j, the sink k, and their connection $j{\rightarrow}k$. Of note, the SE(w) is obtained by lavaan syntax specifying w with the ":=" operator.

2.3 Confirmatory Factor Analysis (CFA) model

The CFA model assumes that the pairwise connected genes j and k are related with a latent variable (LV or factor, F) of unknown common(s) cause(s), and this LV is associated to the disease class (C=0,1), see Figure 2:

$$Y_k = \lambda_k F + U_k; \quad Y_j = \lambda_j F + U_j$$

$$F = \beta C + D \quad \& \quad \text{var}(D) = \phi$$

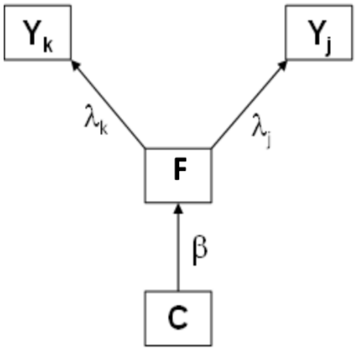

Figure 3: **CFA model.**

For model identification the "common" gene-factor-coefficients, λ's and the variances of U terms (measurement error variables), are fixed values equal to:

$$\lambda = \sqrt{\text{cor}\left(Y_j^{(0)}; (Y_k^{(0)}\right)} \quad \& \quad \text{var}\left(U_k\right) = \text{var}\left(U_j\right) = 1 - \lambda^2$$

For square root computing, if $cor(Y_k; Y_j) < 0$ a negative scaling of Y_j (or Y_k) in $-Y_j$ (or $-Y_k$) is performed. The fitted model with one factor (F), two indicators (Y_j and Y_k) and an exogenous variable (C) has two free parameters, the error variance of the latent variable, ψ, and the regression coefficient, β. Therefore, the CFA model has df=((3x2)/2)–2=1, and can be fitted with **lavaan**. The P-values by the z-test=$\beta/SE(\beta)$ of factor mean differences quantify the group perturbance on the unknown common(s) cause(s) of the covariance of $(Y_k; Y_j)$.

2.4 Fisher's transformation z-to-r correlation method

The correlation method applies the Fisher's r-to z transformation for testing the pairwise difference between the correlation coefficients of linked genes in the input (directed or undirected) interactome, G. The transformation is done in each group, C= (1,0):

$$r_{jk}^{(0)} = \text{cor}\left(Y_j^{(0)}; (Y_k^{(0)}\right) \;\; \& \;\; r_{jk}^{(1)} = \text{cor}\left(Y_j^{(1)}; (Y_k^{(1)}\right)$$

$$z_{jk}^{(0)} = \log\left(\frac{1 + r_{jk}^{(0)}}{1 - r_{jk}^{(0)}}\right) \;\; \& \;\; z_{jk}^{(1)} = \log\left(\frac{1 + r_{jk}^{(1)}}{1 - r_{jk}^{(1)}}\right)$$

The group differences are computed by the classical test, t proposed by Fisher (1915):

$$t_{jk} = \frac{z_{jk}^{(1)} - z_{jk}^{(0)}}{\sqrt{\dfrac{1}{n_1 - 3} + \dfrac{1}{n_0 - 3}}}$$

The P-values of *t's* from $N(0,1)$ distribution measure the group perturbance on the gene-gene correlations.

After P-value computing, the edge weights are defined as inverse of negative logarithm of the P-value, i.e., w= 1/[-log_{10}(P-value)]. In this way, edges with lower P-values (0 to 1) have lower weights (w >0) on a positive continuous range. Intuitively, this weight can be assumed as the perturbance acting on the relationship between two connected genes in the interactome, due to the genotype difference between groups. The lower the P-value (or the w-value), the higher the perturbance. Using the definition of perturbance, we refer to a set of adjacent perturbed edges as a *perturbation route*, originating from a source, passing through a number of connectors, and terminating in a sink node.

3 Evaluation metrics

In the benchmark data analysis, the performance of the state-of-the-art approaches has been evaluated in terms of (i) system perturbation, (ii) disease classifier performance and (iii) COVID-19 gene set/GO enrichment. We also add to the seven extracted CAT modules two reference trees (after CAT conversion): (8) the KEGG "Coronavirus disease - COVID-19" pathway, and (9) the data-driven directed tree extracted from the top 200 DEGs ranking by a Random Forest variable importance procedure with the `randomForest()` function of **randomForest** R package (Breiman, 2001).

(i) Evaluation of system perturbation of extracted CAT subnetworks has been evaluated via `SEMace()` and `SEMgsa()` functions of **SEMgraph**. Firstly, we compute Average Causal Effects (ACEs) between every possible source-sink node pair, using the parent adjustement set procedure (see Table 9 of Supplementary Material), and we report (i) the number of significant paths (P < 0.05 after Bonferroni correction) over the total estimated paths, and (ii) the Bonferroni combination of ACEs' p-values (P=K*min(pvalues)), where K is the total estiamate paths,the lower the value, the better the score. Then, we perform a Gene Set Analysis (GSA) on CAT modules and we report (iii) node activation and node inhibition P-values (P+ and P-, respectively) through a Bonferroni statistics ($P = 2 * min(P+; P-)$), and (ii) the number of DEGs, i.e. differential expression genes with P-values < 0.05 after Benjamini-Hochberg (BH) correction.

(ii) Disease classifier performance was carried out by a penalized Fisher's Discriminant Analysis (pFDA) with the `PenalizedLDA()` function of **PenalizedLDA** R package Witten and Tibshirani (2011) to identify genes in the extracted subnetworks able to discriminate between groups. Specifically, pFDA tries a discriminant projection (a discriminant variable), $a^T x = \Sigma_j a_j x_j$ in a lower dimensional space such that the ratio of between-class variance and within class variance is maximized, subject to an additional l1 (lasso)-constraint on the weights, $a's$. This constraint ensures that some discriminant weights, a_j will be estimated as exactly zero and the corresponding variable, will not be contribute to the discriminant variable. Then, the FDA threshold $d = (a^T\overline{x}_0 + a^T\overline{x}_1)/2$ was defined to classify the patients as case if $a^T x - d > 0$ or as non-case, vice versa.
To avoid model over-performance on a specific dataset, and consequent loss of classification generality, and reproducibility, we performed a K-fold cross-validation analysis, with $K = 5$. At each iteration, K-1 partitions were merged into one and used for the learning process (training step), while the K-th left out partition (i.e., the validation set) was used to predict the outcome (i.e., the diagnostic class). The 2x2 frequency table (i.e. confusion matrix) was obtained at each iteration of the K-fold cross-validation, and the classical performance indices of the FDA classifier (sensitivity, specificity, and accuracy). Let TP be the true positives from the 2x2 confusion table, FP be the false positive, TN be the true negative, and FN be the false negative. Then, Se= TP/(TP+FN), Sp=TN/(TN+FP), and Ac=(TP+TN)/n, where n is the total sample, and the confusion table is computed both by averaging the indices of K 2x2 tables and by using the overall 2x2 table over the K iterations.

(iii) 3033 genes which were contained both in COVID-19 gene expression profiles and KEGG network were included in the subsequent network analysis. In addition, 245 genes related to COVID-19 disease were obtained from the collection of diseases-related genes of Feng *et al.* (2022). This data based comprehensively included genes collected from searches against OMIM (), KMDB/Mutation-View (), DisGeNET database (), and NCBI database Tatusova *et al.* (2016). Non-matching genes derived from an updated version of the databases were added, resulting in 278 total genes. Among the 278 genes, 92 were included in the 3033 genes of the benchmark expression dataset. Then, we extracted the GO terms related to the 92 COVID-19 reference genes, resulting in a total of 1099 recovered GO terms.
We perform an assessment of enrichment performance, both on benchmark and simulated data, looking at precision, recall and F1 score. To this goal, the genes (or the GO terms) are separated into two groups, Foreground Genes FG (or Foreground GO terms, FGO) and Background Genes BG (or Background GO

terms). The FG (FGO) are the reference 92 COVID-19 genes (1099 GO terms), while, for simulated data, FG genes are artificially differentially expressed. Let TP and the FP the number of FG and BG present in the active modules, respectively, and the FN are the number of missing FG (i.e. the FG that were not retrieved). Then, Pre = TP/(TP+FP), Rec = TP/(TP+FN), and F1 = 2*(Prec*Rec)/(Pre+Rec) have been computed, taking the average over 100 simulation runs for simulated data.

(iv) We simulate five datases from multivariate normal distributions. Each dataset consists of 500 genes and 20 samples and the condition-specific sub-network for case datasets 1, 2, 3 consisted of 50 genes while for case dataset 4 consisted of 40 genes. Specifically:

- Control group: $\mu = \rho = 0$ for all genes (to be compared with each of the four case sets for the identification of the optimal sub-network).
- Case set 1 (both differential expression and differential correlation): Gene 1 to Gene 50 have $\mu = 0.75$, and $\rho = 0.6$ between each gene pair; the other 450 genes have $\mu = \rho = 0$.
- Case set 2 (only differential expression, no differential correlation): Gene 1 to Gene 50 have $\mu = 0.75$, and $\rho = 0$ between each gene pair; the other 450 genes have $\mu = \rho = 0$.
- Case set 3 (differential correlation and differential expression with both up and down regulation): Gene 1 to Gene 25 have $\mu = 0.75$, and $\rho = 0.6$ between each pair; Gene 26 to Gene 50 have $\mu = -0.75$ and $\rho = 0.6$ between each pair of them; $\rho = -0.6$ between any gene from 1 to 25 and any gene from 26 to 50. The other 450 genes have $\mu = 0$ and $\rho = 0$.
- Case set 4: 10 genes from each of set 2, set 3, set 4 and set 5, the other 460 genes from set 1 (mixed pattern of differential expression and differential correlation).

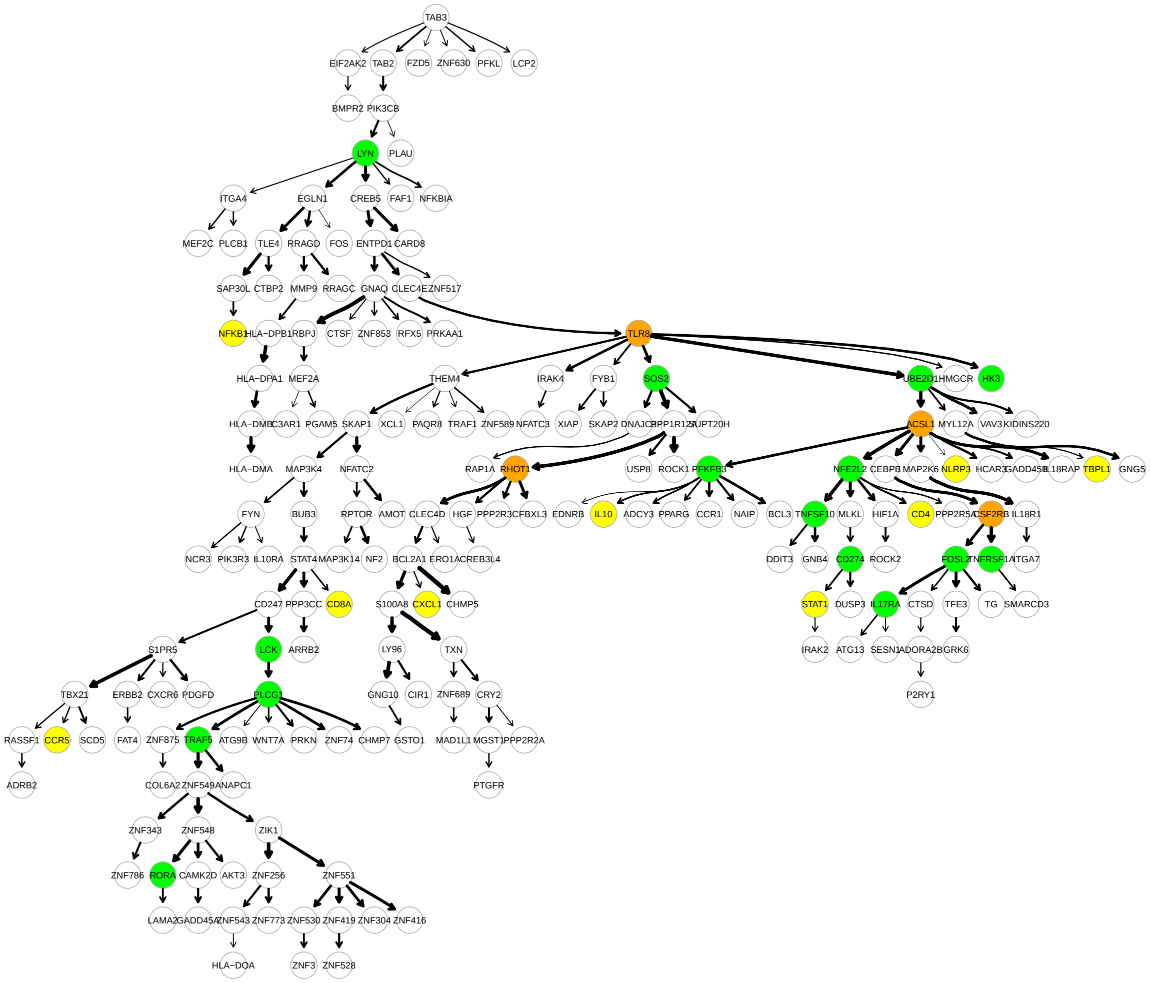

Figure 4: **BioNet recovered subnetwork from benchmark data analysis.** Nodes in the recovered subnetworks are coloured in yellow if they represent COVID-19 related genes while the cluster summarised by the prototype is coloured in orange if it contains at least one COVID-19 related gene (in green otherwise). The width of edges shows the strength of correlation coefficient of pairs of interacting nodes.

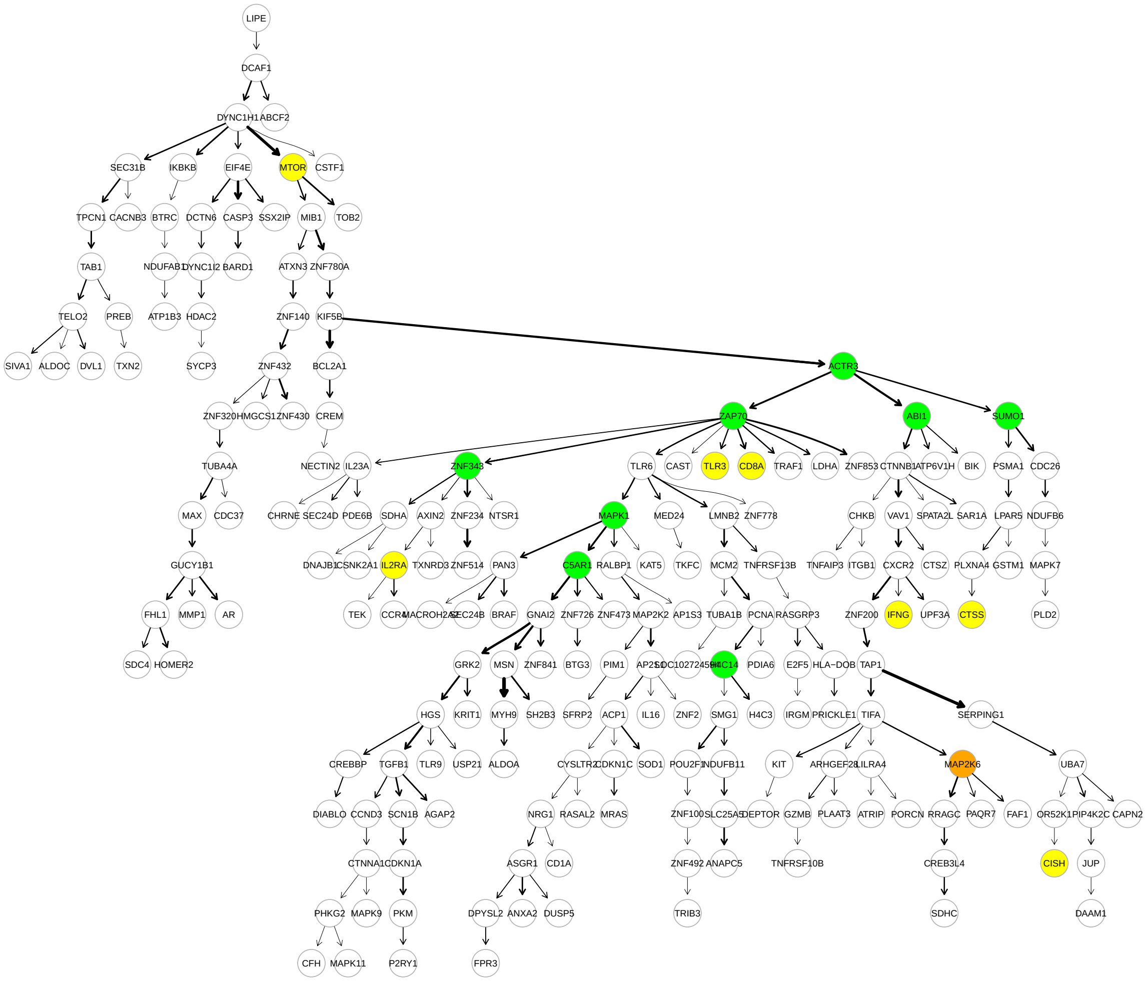

Figure 5: **COSINE recovered subnetwork from benchmark data analysis.** Nodes in the recovered subnetworks are coloured in yellow if they represent COVID-19 related genes while the cluster summarised by the prototype is coloured in orange if it contains at least one COVID-19 related gene (in green otherwise). The width of edges shows the strength of correlation coefficient of pairs of interacting nodes.

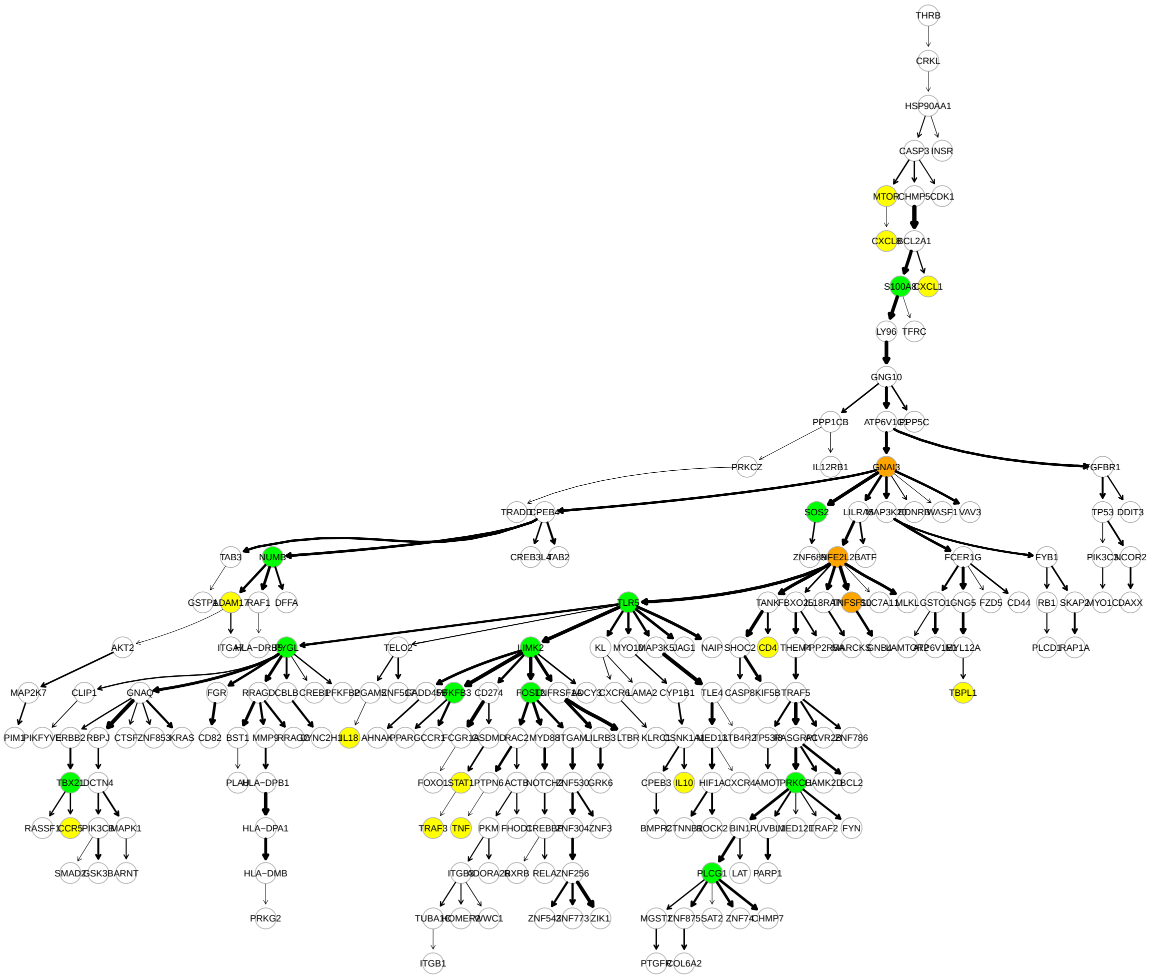

Figure 6: **pathfindeR recovered subnetwork from benchmark data analysis.** Nodes in the recovered subnetworks are coloured in yellow if they represent COVID-19 related genes while the cluster summarised by the prototype is coloured in orange if it contains at least one COVID-19 related gene (in green otherwise). The width of edges shows the strength of correlation coefficient of pairs of interacting nodes.

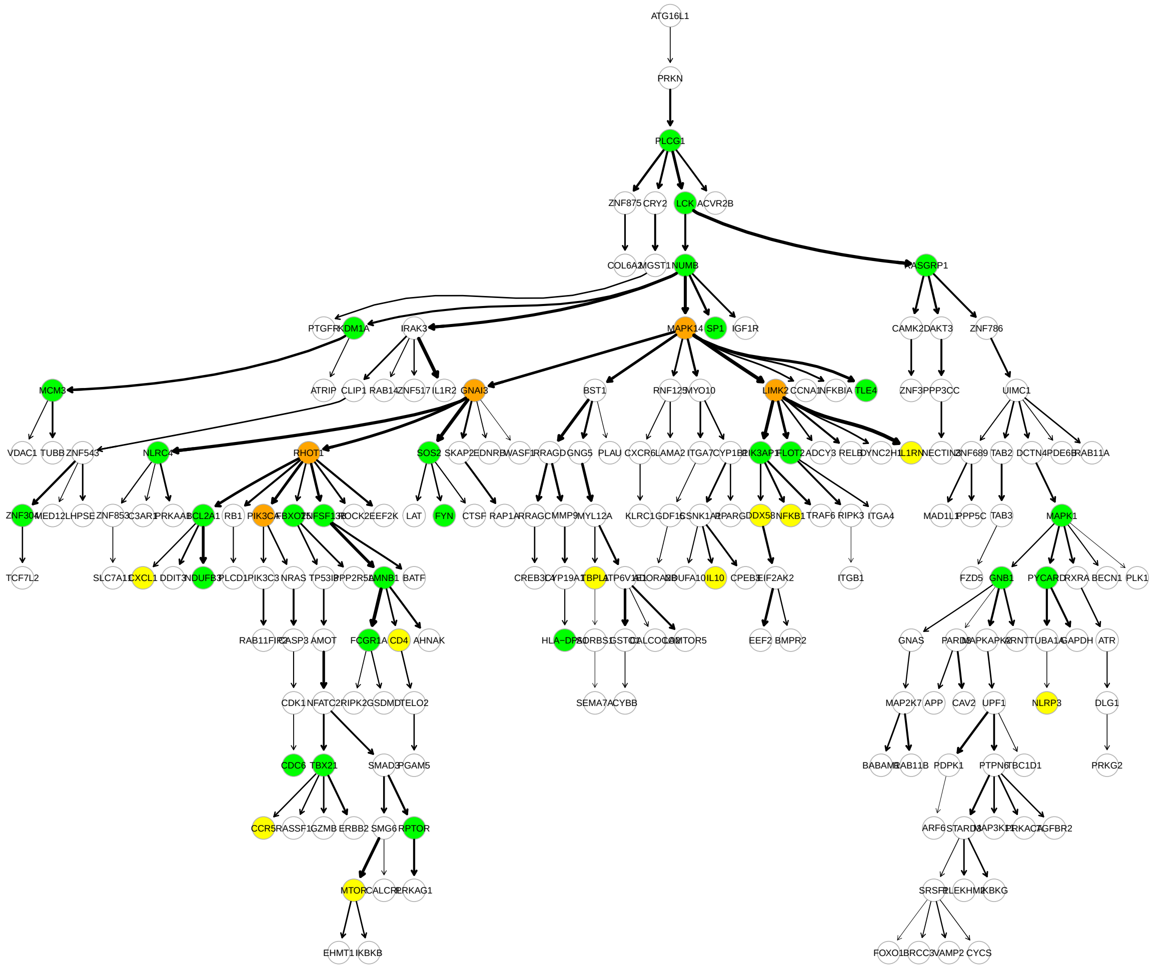

Figure 7: **ST recovered subnetwork from benchmark data analysis.** Nodes in the recovered subnetworks are coloured in yellow if they represent COVID-19 related genes while the cluster summarised by the prototype is coloured in orange if it contains at least one COVID-19 related gene (in green otherwise). The width of edges shows the strength of correlation coefficient of pairs of interacting nodes.

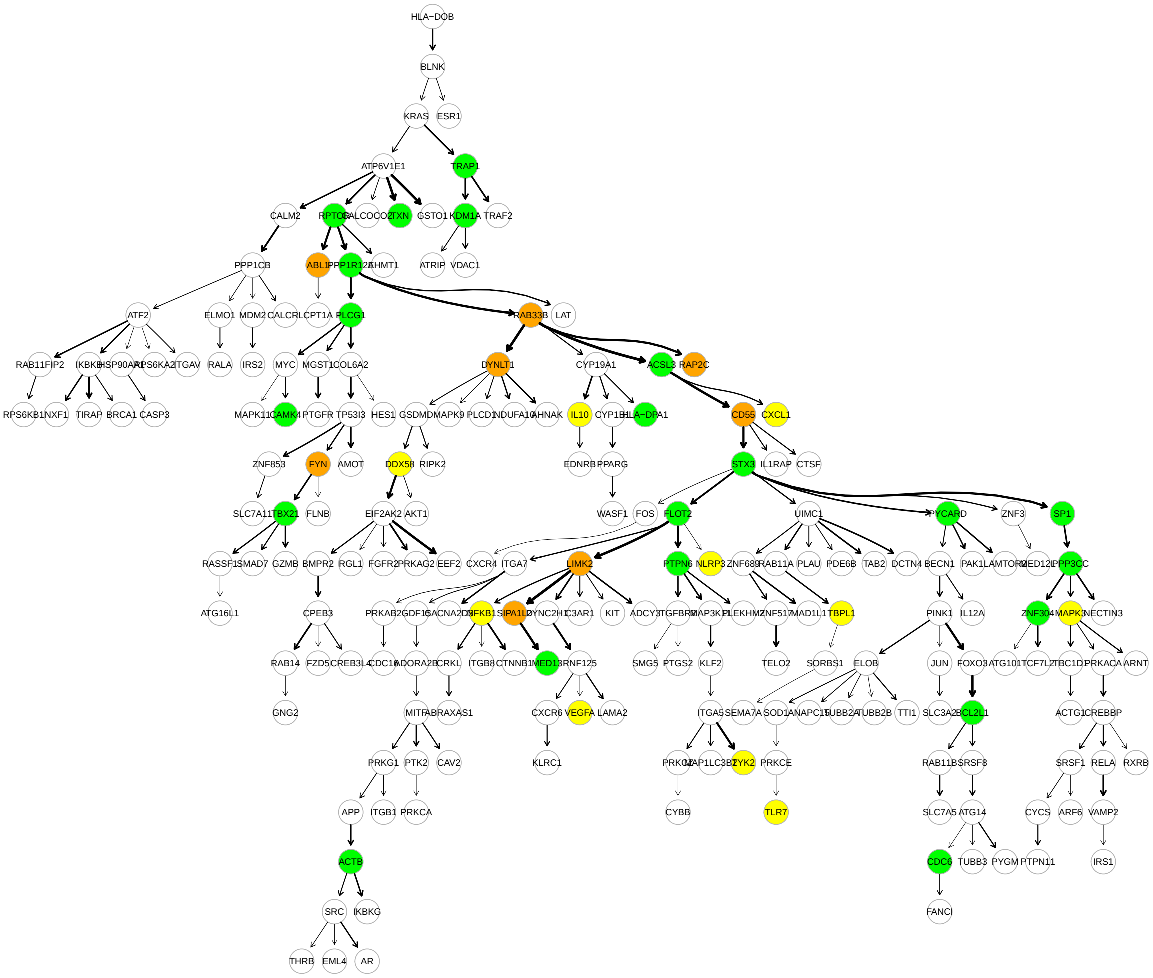

Figure 8: **STr2z recovered subnetwork from benchmark data analysis.** Nodes in the recovered subnetworks are coloured in yellow if they represent COVID-19 related genes while the cluster summarised by the prototype is coloured in orange if it contains at least one COVID-19 related gene (in green otherwise). The width of edges shows the strength of correlation coefficient of pairs of interacting nodes.

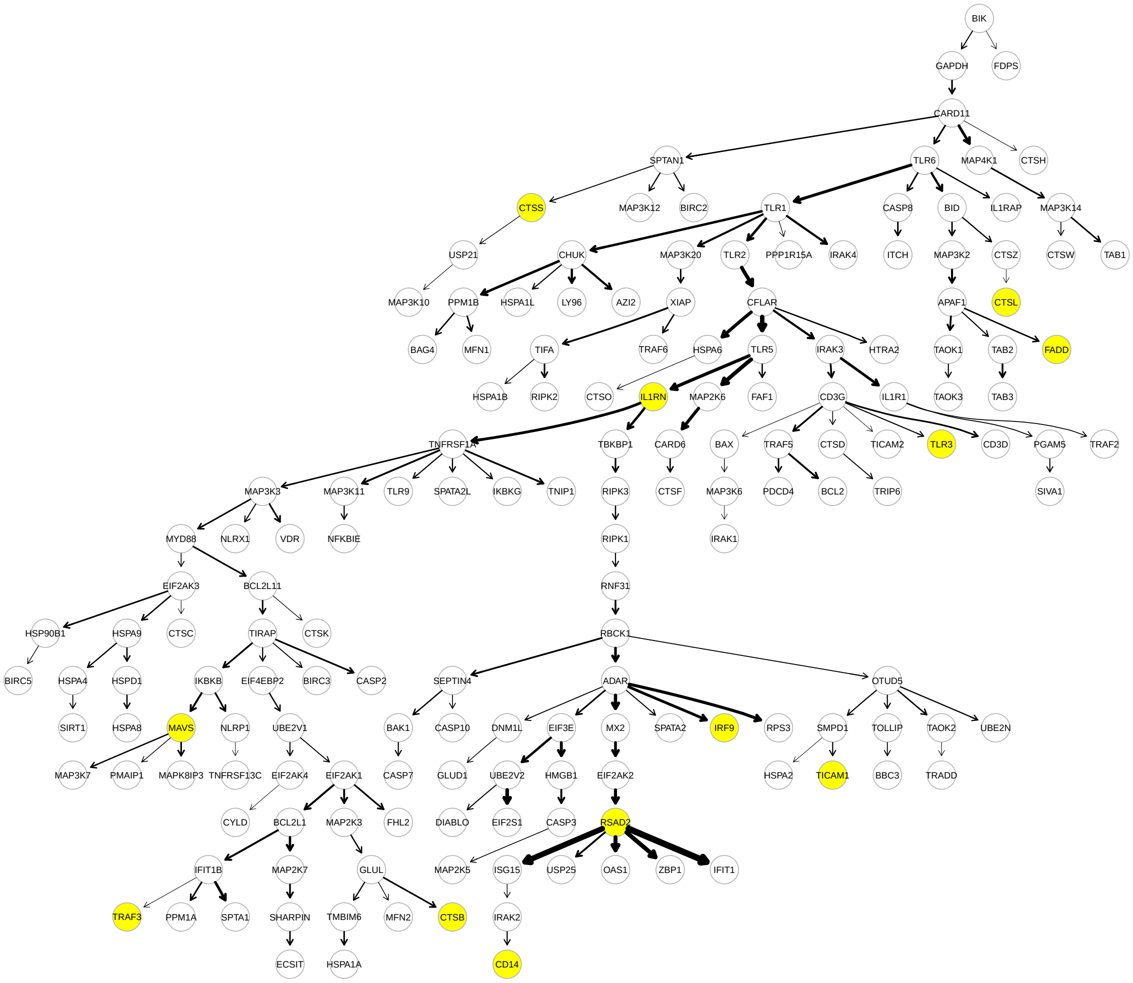

Figure 9: **WalktrapGM (with iPINBPA weights) recovered subnetwork from benchmark data analysis.** Nodes in the recovered subnetworks are coloured in yellow if they represent COVID-19 related genes while the cluster summarised by the prototype is coloured in orange if it contains at least one COVID-19 related gene (in green otherwise). The width of edges shows the strength of correlation coefficient of pairs of interacting nodes.

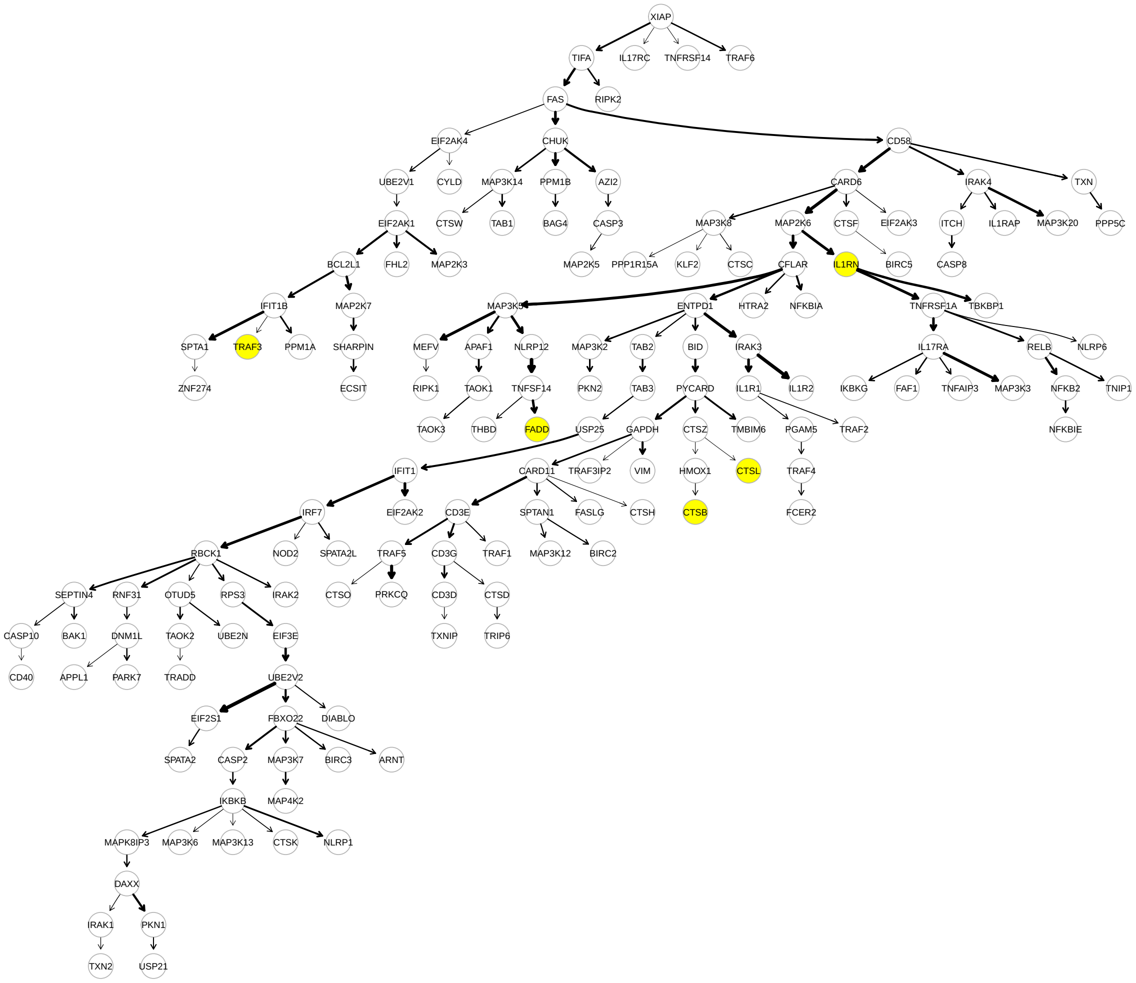

Figure 10: **WalktrapGM (with FC weights) recovered subnetwork from benchmark data analysis.** Nodes in the recovered subnetworks are coloured in yellow if they represent COVID-19 related genes while the cluster summarised by the prototype is coloured in orange if it contains at least one COVID-19 related gene (in green otherwise). The width of edges shows the strength of correlation coefficient of pairs of interacting nodes.

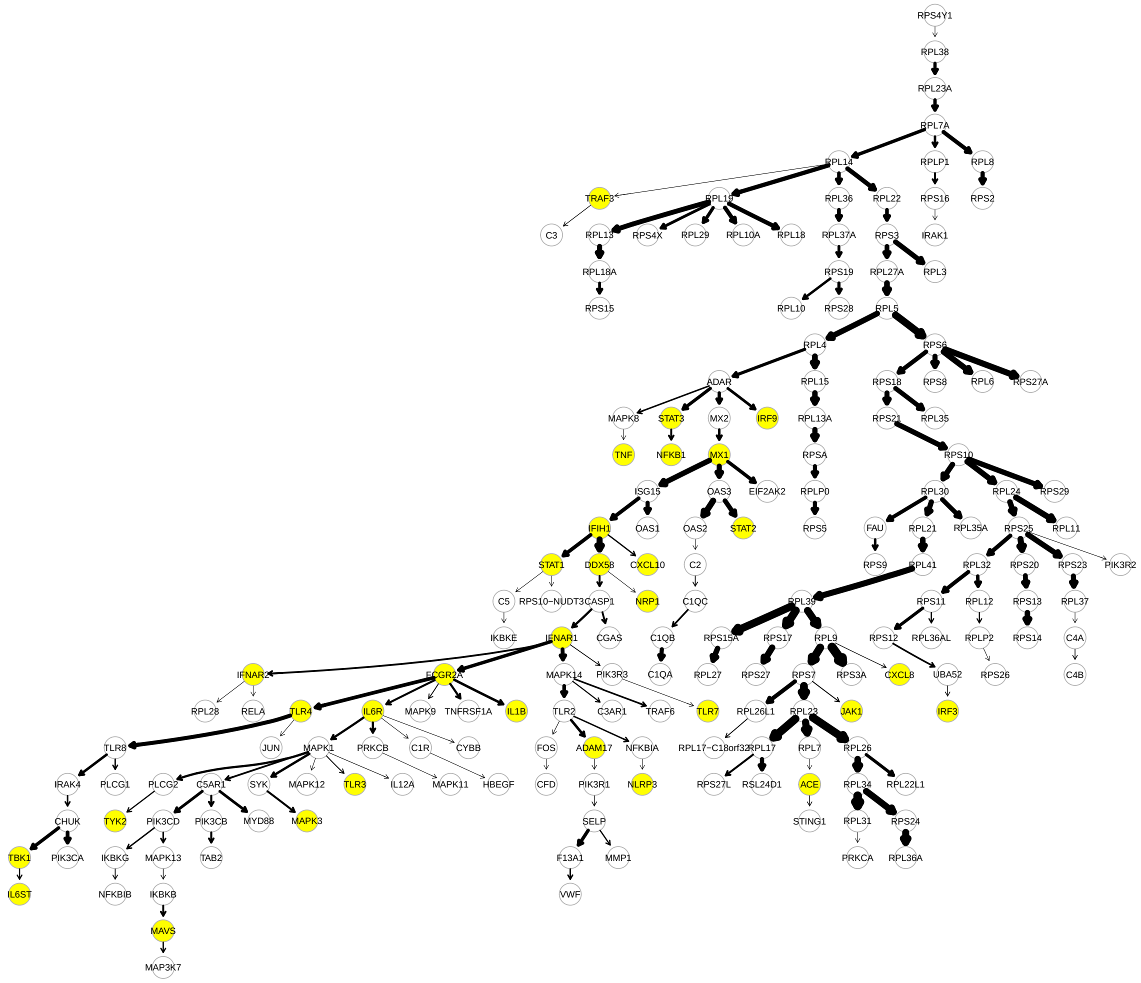

Figure 11: **KEGG "Coronavirus disease - COVID-19" pathway" recovered subnetwork from benchmark data analysis.** Nodes in the recovered subnetworks are coloured in yellow if they represent COVID-19 related genes while the cluster summarised by the prototype is coloured in orange if it contains at least one COVID-19 related gene (in green otherwise). The width of edges shows the strength of correlation coefficient of pairs of interacting nodes.

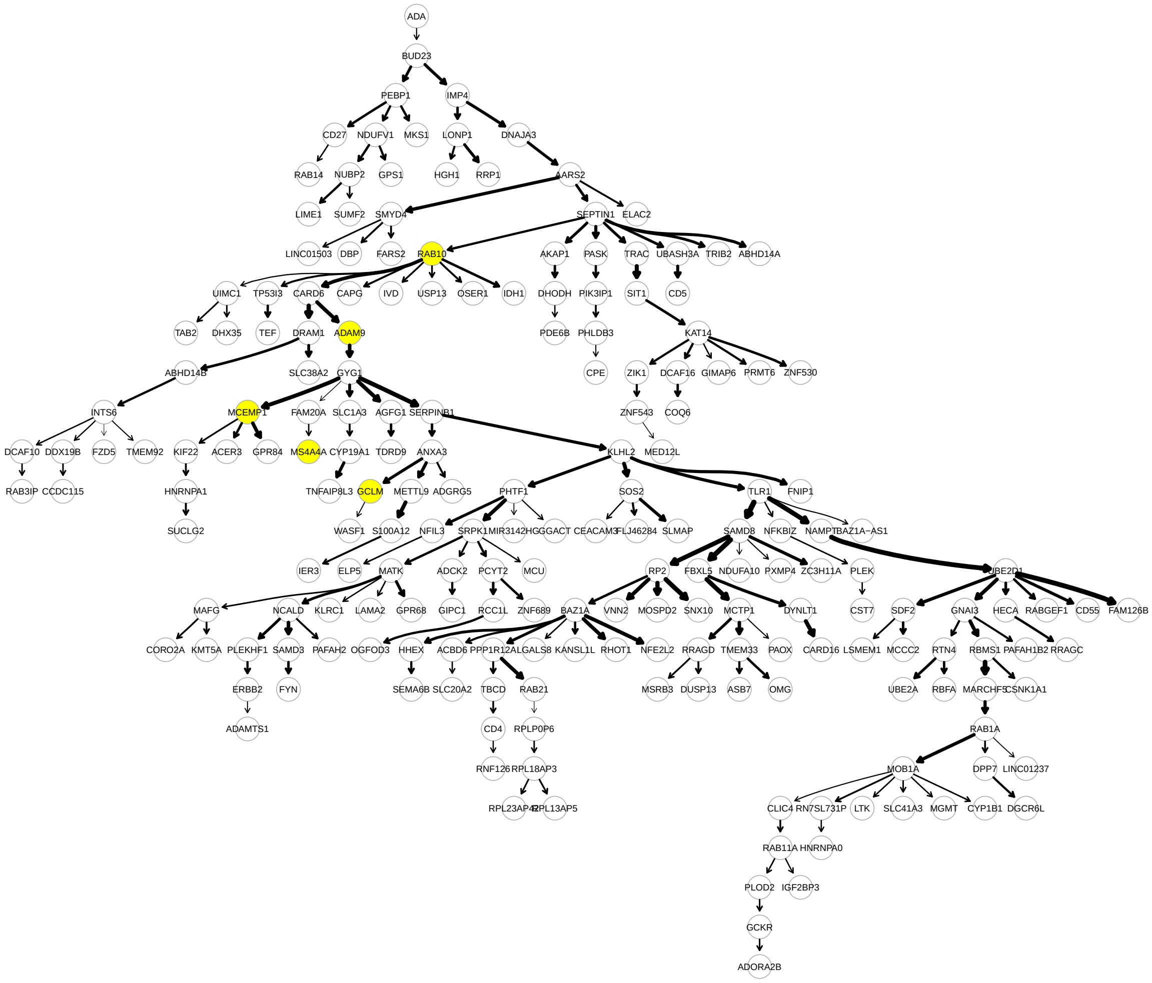

Figure 12: **Random forest COVID-19 recovered subnetwork from benchmark data analysis.** Nodes in the recovered subnetworks are coloured in yellow if they represent COVID-19 related genes while the cluster summarised by the prototype is coloured in orange if it contains at least one COVID-19 related gene (in green otherwise). The width of edges shows the strength of correlation coefficient of pairs of interacting nodes.

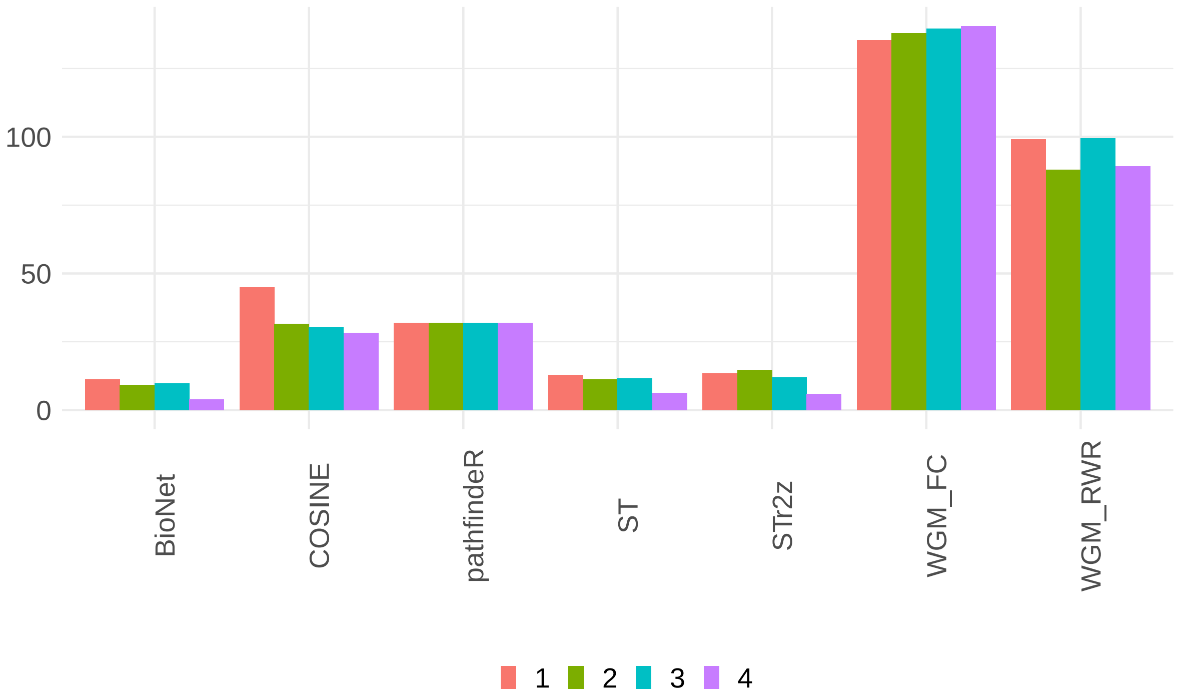

Figure 13: **Average size of the recovered subnetwork for each method on simulated data.** Nodes in the recovered subnetworks are coloured in yellow if they represent COVID-19 related genes while the cluster summarised by the prototype is coloured in orange if it contains at least one COVID-19 related gene (in green otherwise). The width of edges shows the strength of correlation coefficient of pairs of interacting nodes.

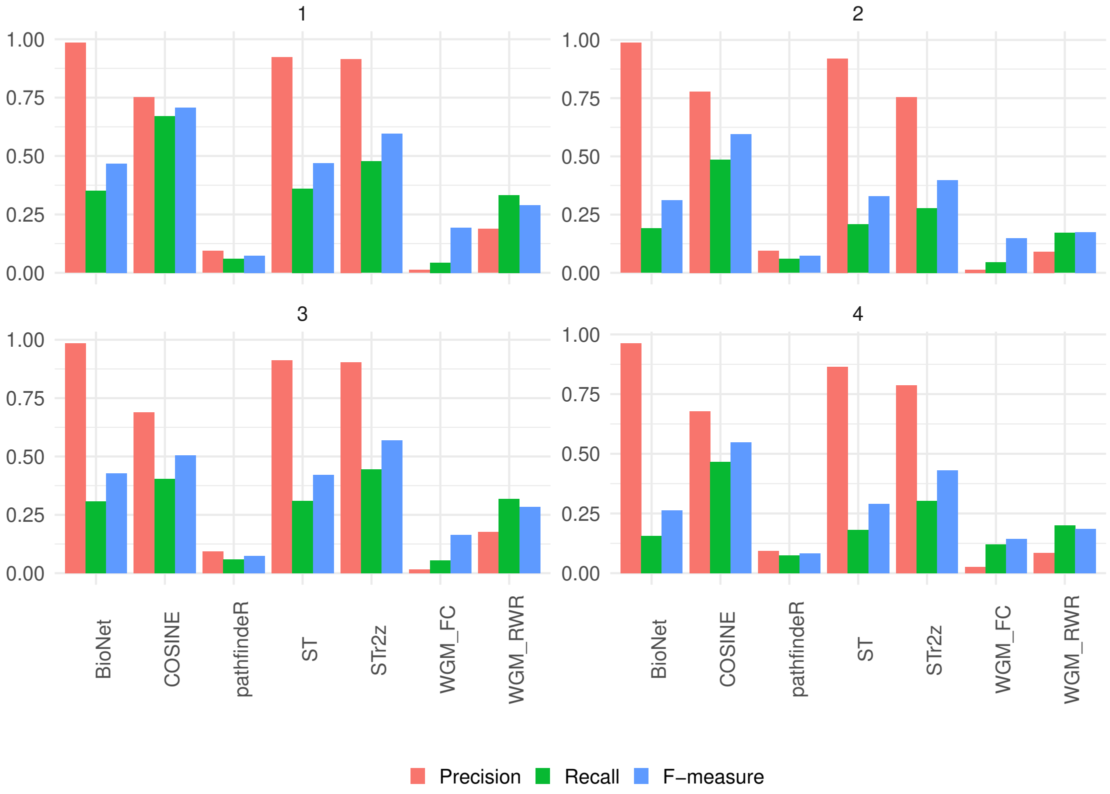

Figure 14: **Precision, recall and F1-score on simulated data.** The mean over 100 simulation runs of precision, recall and F1- score are displayed for each method and for each case dataset. Results show high precision score for ST and STsem, just below the precision of BioNet.

Table 1: **Recovered genes/GO and selected COVID-19 related genes/GO for the nine recovered subnetworks from benchmark data analysis.**

method	all_genes	C19_genes	all_GO	C19_GO
BioNet	193	13	862	440
COSINE	206	8	391	184
pathfindeR	205	15	1708	703
ST	192	15	1315	535
STr2z	204	18	1855	650
WGM_RWR	166	12	872	432
WGM_FC	155	5	756	397
KEGG_C19	183	31	815	507
RF_C19	200	5	0	0

Table 2: **Jaccard similarity indices between the node sets of the nine recovered subnetworks from benchmark data analysis.**

	BioNet	COSINE	pathfindeR	ST	STr2z	WGM_RWR	WGM_FC	KEGG	RF
BioNet	1.00	0.03	0.28	0.22	0.12	0.04	0.06	0.04	0.05
COSINE	0.03	1.00	0.04	0.03	0.04	0.04	0.04	0.02	0.00
pathfindeR	0.28	0.04	1.00	0.31	0.19	0.05	0.05	0.03	0.06
ST	0.22	0.03	0.31	1.00	0.35	0.05	0.06	0.04	0.07
STr2z	0.12	0.04	0.19	0.35	1.00	0.03	0.04	0.05	0.05
WGM_RWR	0.04	0.04	0.05	0.05	0.03	1.00	0.51	0.06	0.01
WGM_FC	0.06	0.04	0.05	0.06	0.04	0.51	1.00	0.04	0.01
KEGG	0.04	0.02	0.03	0.04	0.05	0.06	0.04	1.00	0.00
RF	0.05	0.00	0.06	0.07	0.05	0.01	0.01	0.00	1.00

Table 3: **Gene enrichment metrics for all ST methods on benchmark data analysis.**

method	all_genes	C19_genes	GenePre	GeneRec	GeneF1
ST	192	15	0.08	0.16	0.11
STone	201	15	0.07	0.16	0.10
STr2zN	203	16	0.08	0.17	0.11
STr2z	204	18	0.09	0.20	0.12
STsem	200	16	0.08	0.17	0.11
STcov	195	18	0.09	0.20	0.13
STcfa	202	16	0.08	0.17	0.11

Table 4: **GO enrichment metrics for all ST methods on benchmark data analysis.**

method	all_GO	C19_GO	GOPre	GORec	GOF1
ST	1315	535	0.41	0.49	0.44
STone	1759	613	0.35	0.56	0.43
STr2zN	1493	554	0.37	0.50	0.43
STr2z	1855	650	0.35	0.59	0.44
STsem	1853	665	0.36	0.61	0.45
STcov	1710	634	0.37	0.58	0.45
STcfa	1660	602	0.36	0.55	0.44

Table 5: **Perturbation metrics for all ST methods on benchmark data analysis.**

method	graph	h	tree	ACEs(%)	PVAL(E)	No. DEGs	PVAL(V)
ST	(396;395)	0.16	(192;191)	64	5.41e-06	103	4.91E-10
STone	(389;388)	0.16	(201;200)	0	5.86e+00	106	3.11E-13
STr2zN	(435;434)	0.16	(203;202)	0	5.76e-01	116	6.28E-13
STr2z	(459;458)	0.2	(204;203)	22	1.55e-05	94	3.17E-13
STsem	(397;396)	0.15	(200;199)	0	7.42e-02	110	3.08E-13
STcov	(439;438)	0.2	(195;194)	10	2.45e-02	99	4.74E-07
STcfa	(410;409)	0.16	(202;201)	13	5.49e-02	110	3.11E-13

Table 6: **Disease classifier performance metrics for all ST methods on benchmark data analysis.**

	No. genes	No. zero	Se	Sp	Acc
ST	192	46	0.96	0.87	0.93
STone	201	69	0.96	0.87	0.93
STr2zN	194	56	0.96	0.87	0.93
STr2z	204	87	0.96	0.87	0.93
STsem	200	55	0.96	0.87	0.93
STcov	195	78	0.96	0.87	0.93
STcfa	199	51	0.96	0.87	0.93

Table 7: **Jaccard similarity indices between the node sets for all ST methods on benchmark data analysis.**

	ST	STone	STr2z	STr2zN	STsem	STcov	STcfa
ST	1.00	0.58	0.74	0.35	0.54	0.39	0.63
STone	0.58	1.00	0.57	0.38	0.66	0.45	0.60
STr2zN	0.74	0.57	1.00	0.35	0.51	0.40	0.65
STr2z	0.35	0.38	0.35	1.00	0.37	0.47	0.34
STsem	0.54	0.66	0.51	0.37	1.00	0.41	0.57
STcov	0.39	0.45	0.40	0.47	0.41	1.00	0.40
STcfa	0.63	0.60	0.65	0.34	0.57	0.40	1.00

Table 8: **Significant average causal effects (ACEs) between source-sink pairs as obtained from SEMace function while testing for perturbation with SEMpath.**

pathL	sink <- source	d_est	d_se	d_z	pvalue	d_lower	d_upper
9	DDIT3 <- ATG16L1	0.90	0.26	3.50	0	0.39	1.40
9	NDUFA10 <- ATG16L1	-0.92	0.24	-3.79	0	-1.39	-0.44
9	CPEB3 <- ATG16L1	0.88	0.24	3.67	0	0.41	1.34
8	MAD1L1 <- ATG16L1	-0.93	0.20	-4.74	0	-1.31	-0.54
9	SLC7A11 <- ATG16L1	0.79	0.22	3.66	0	0.37	1.21
6	IL1R2 <- ATG16L1	0.80	0.19	4.11	0	0.42	1.18
13	CCR5 <- ATG16L1	-0.87	0.21	-4.23	0	-1.27	-0.47
6	NFKBIA <- ATG16L1	1.02	0.22	4.65	0	0.59	1.45
6	TLE4 <- ATG16L1	1.00	0.25	4.05	0	0.51	1.48
10	EEF2 <- ATG16L1	-0.94	0.25	-3.82	0	-1.42	-0.46
10	AHNAK <- ATG16L1	-0.80	0.22	-3.68	0	-1.23	-0.37
8	PPARG <- ATG16L1	0.87	0.24	3.68	0	0.40	1.33
7	RELB <- ATG16L1	0.83	0.23	3.62	0	0.38	1.27
7	DYNC2H1 <- ATG16L1	-0.83	0.20	-4.14	0	-1.22	-0.43
7	IL1RN <- ATG16L1	0.90	0.24	3.78	0	0.43	1.36
8	FYN <- ATG16L1	-0.92	0.17	-5.44	0	-1.25	-0.59
8	CTSF <- ATG16L1	-0.69	0.17	-4.02	0	-1.03	-0.35
6	ZNF3 <- ATG16L1	-0.72	0.19	-3.74	0	-1.10	-0.34
9	PPP2R5A <- ATG16L1	0.84	0.23	3.65	0	0.39	1.29

Table 9: **Average simulation results (over 100 runs) for the seven subnetwork detection methods for each case dataset.**

method	set	size_mean	precision_mean	recall_mean	f1_mean
BioNet	1	11.29	0.98	0.35	0.47
BioNet	2	9.30	0.99	0.19	0.31
BioNet	3	9.78	0.99	0.31	0.43
BioNet	4	3.93	0.96	0.16	0.26
COSINE	1	44.95	0.75	0.67	0.71
COSINE	2	31.61	0.78	0.49	0.60
COSINE	3	30.24	0.69	0.40	0.51
COSINE	4	28.37	0.68	0.47	0.55
pathfindeR	1	32.00	0.09	0.06	0.07
pathfindeR	2	32.00	0.09	0.06	0.07
pathfindeR	3	32.00	0.09	0.06	0.07
pathfindeR	4	32.00	0.09	0.08	0.08
ST	1	12.92	0.92	0.36	0.47
ST	2	11.24	0.92	0.21	0.33
ST	3	11.62	0.91	0.31	0.42
ST	4	6.35	0.87	0.18	0.29
STr2z	1	13.51	0.92	0.48	0.60
STr2z	2	14.77	0.76	0.28	0.40
STr2z	3	12.05	0.90	0.44	0.57
STr2z	4	5.99	0.79	0.30	0.43
WGM_RWR	1	99.13	0.19	0.33	0.29
WGM_RWR	2	87.93	0.09	0.17	0.17
WGM_RWR	3	99.44	0.18	0.32	0.28
WGM_RWR	4	89.27	0.08	0.20	0.19
WGM_FC	1	135.40	0.01	0.04	0.19
WGM_FC	2	137.97	0.01	0.05	0.15
WGM_FC	3	139.59	0.02	0.06	0.16
WGM_FC	4	140.52	0.03	0.12	0.14

References

Breiman, L. (2001). Random forests. *Machine Learning*, **45**, 5–32.

Dittrich, M. T. *et al.* (2008). Identifying functional modules in protein–protein interaction networks: an integrated exact approach. *Bioinformatics*, **24**(13), i223–i231.

Feng, S. *et al.* (2022). Potential genes associated with covid-19 and comorbidity. *International journal of medical sciences*, **19**, 402–415.

Fisher, R. A. (1915). Frequency distribution of the values of the correlation coefficient in samples from an indefinitely large population. *Biometrika*, **10**(4), 507–521.

Ideker, T. *et al.* (2002). Discovering regulatory and signalling circuits in molecular interaction networks. *Bioinformatics*, **18**(suppl_1), $S233 - -S240$.

Kou, L. T. *et al.* (1981). A fast algorithm for steiner trees. *Acta Informatica*, **15**, 141–145.

Köhler, S. *et al.* (2008). Walking the interactome for prioritization of candidate disease genes. *American journal of human genetics*, **82**, 949–58.

Ma, H. *et al.* (2011). COSINE: COndition-SpecIfic sub-NEtwork identification using a global optimization method. *Bioinformatics*, **27**(9), 1290–1298.

Petrochilos, D. *et al.* (2013). Using random walks to identify cancer-associated modules in expression data. *BioData mining*, **6**, 17.

Tatusova, T. *et al.* (2016). Ncbi prokaryotic genome annotation pipeline. *Nucleic Acids Research*, **44**, 6614–24.

Ulgen, E. *et al.* (2019). pathfindr: An r package for comprehensive identification of enriched pathways in omics data through active subnetworks. *Frontiers in Genetics*, **10**.

Wang, L. *et al.* (2015). ipinbpa: an integrative network-based functional module discovery tool for genome-wide association studies. volume 20.

Witten, D. M. and Tibshirani, R. (2011). Penalized classification using fisher's linear discriminant. *Journal of the Royal Statistical Society. Series B (Statistical Methodology)*, **73**(2), 753–772.
